# Reaction Medium as an Architect of Nanocrystal Superlattices

**DOI:** 10.1021/jacs.6c07859

**Published:** 2026-07-15

**Authors:** Seungho Lee, Daniel M. Balazs, Aiswarya Rayaroth, Sharona Horta, Carl P. Goodrich, Michael Engel, Ihor Cherniukh, Maria Ibáñez

**Affiliations:** † Institute of Science and Technology Austria (ISTA), Klosterneuburg 3400, Austria; ‡ Institute for Multiscale Simulation, Friedrich-Alexander-Universität Erlangen-Nürnberg;, Erlangen 91058, Germany

## Abstract

Nanocrystal superlattices are commonly formed by changing concentration, solvent conditions, or particle surface chemistry. Although effective, these approaches alter multiple contributions to the interparticle potential simultaneously, making it difficult to isolate the interactions responsible for ordering or to control assembly in chemically complex environments. Here, we show that oligomeric species present in a nanocrystal reaction medium drive superlattice formation through a depletion mechanism. Using PbTe nanocrystals as a model system, we identify Pb–oleate oligomers in the crude reaction mixture, characterize their solution structure, and quantify their contribution to the interparticle potential, establishing depletion as the dominant short-range interaction governing spontaneous body-centered cubic superlattice formation. We then confirm the depletion origin of ordering by showing that varying depletant concentration predictably shifts the order–disorder boundary and produces a thermally reversible transition between dispersed and ordered states — behavior that is inconsistent with van der Waals or ligand-mediated mechanisms but is a direct consequence of depletion control. Having established and validated the mechanism, we demonstrate that the same depletion framework can be deliberately activated in purified dispersions and transferred across nanocrystal systems of different composition and shape, including anisotropic and binary assemblies. These results establish precursor-derived depletion as a general and chemically grounded mechanism for nanocrystal superlattice formation, and show that collective ordering can be programmed through the surrounding medium rather than through particle surface modification.

## Graphical abstract

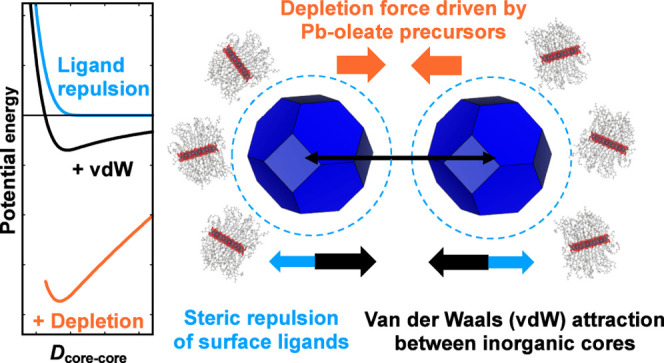

## Introduction

Colloidal synthesis of inorganic nanocrystals (NCs) capped by long-chain organic ligands offers precise control over their size, shape, crystal phase, composition, and surface chemistry,^1^ enabling their use as well-defined building blocks for hierarchical materials.^2^ When assembled into periodic arrangements, NCs can give rise to collective optical,^3,4^ mechanical,^5^ electrochemical,^6,7^ electrical,^8^ and thermal properties that are inaccessible in disordered NC arrays.^9^ Realizing these properties requires not only forming ordered superlattices but understanding and controlling the interactions that stabilize them.

In sterically stabilized NCs, ordering is governed by a balance of van der Waals attraction, ligand-induced steric repulsion, and ligand–solvent interactions.^2,10^ Entropic contributions, including the free-volume gain of the NC ensemble^11–14^ and the configurational freedom of bound ligands,^15,16^ further shape the effective interparticle potential. For ordered superlattice formation, this potential typically features a shallow attractive minimum beyond the repulsive core, enabling NCs to associate while retaining sufficient freedom to rearrange into crystalline structures rather than aggregate irreversibly.^2^ In practice, this balance is typically achieved by adjusting NC concentration,^17^ temperature,^17,18^ solvent quality,^19,20^ or evaporation rate.^21^ However, these parameters simultaneously affect multiple interaction terms and kinetic processes, coupling changes in attraction strength, ligand solvation, and particle mobility.^19,21–25^ As a result, whether ordering is achieved and whether it remains reversible can depend on the preparation pathway, because the same knobs reshape both the interparticle potential and the kinetic barriers for rearrangement.

Similar ordering behavior has also been observed under chemically complex conditions, including during NC synthesis or cooling of reaction mixtures.^26–35^ These observations span a wide range of material systems and have attracted considerable attention, yet their mechanistic origin remains poorly understood. Assembly under synthesis conditions is typically attributed to van der Waals attraction between sufficiently large NCs, changes in ligand solvation during cooling, or shifts in solvent quality. However, all these mechanisms likewise rely on the same coupled interaction landscape and therefore face the same fundamental interpretive limitations. Crucially, none of these accounts identifies a specific chemical species responsible for driving assembly. The connection between reaction medium composition and collective NC organization therefore remains undefined, making it difficult to predict, control, or deliberately exploit ordering in chemically realistic environments.

Depletion attraction offers a distinct and largely overlooked route to understanding and controlling this behavior. Depletion is an entropic interaction arising from the exclusion of surrounding molecular or oligomeric species, so-called depletants, from the gap between approaching particles. It generates a short-range attractive potential whose strength and range are governed by the size, concentration of the depletants, independently of NC surface chemistry and solvent quality.^36^ Depletion interactions have previously been exploited in NC systems using deliberately introduced polymers or surfactants to induce postsynthetic assembly, phase separation, and size- or shape-selective purification.^12,13,37,38^ However, whether species already present in synthesis environments (precursor complexes, ligand oligomers, reaction intermediates) can function as intrinsic depletants, and whether this mechanism accounts for the spontaneous ordering widely observed during synthesis, has not been established.

Here we demonstrate that precursor-derived oligomeric species act as intrinsic depletants in NC reaction media, providing for the first time a chemically specific and quantitative account of superlattice formation under synthesis-realistic conditions. Using PbTe NCs as a model system, we identify Pb–oleate oligomers present in the reaction medium, characterize their solution structure by small-angle X-ray scattering (SAXS), and show that they generate a depletion attraction sufficient to account for the spontaneous formation of 3D body-centered cubic (BCC) superlattices. We then validate this mechanistic assignment by demonstrating that depletant concentration serves as a single, predictive control parameter for the order–disorder boundary, producing thermally reversible assembly and disassembly that is inconsistent with van der Waals or ligand-mediated mechanisms. Finally, we show that the same depletion framework can be deliberately transferred to purified dispersions and applied across NC systems of different composition and shape, including anisotropic and binary assemblies. Together, these results establish depletion as a general mechanism underlying a broad class of synthesis-associated ordering phenomena and provide a rational framework for programming collective organization through the chemical environment surrounding the NCs.

## Results and Discussion

As a model system to investigate depletion-driven NC assembly, we use PbTe NCs synthesized by combining a mixture of lead oleate (PbOA_2_) and oleic acid (OA) with trioctylphosphine telluride (TOP-Te) in hexadecane (HDE), as depicted in Figure 1a. Upon cooling, the color of the crude solution changes from clear yellow-brown to turbid black, indicating loss of colloidal stability (Figure S1). The resulting aggregation in the crude solution was analyzed by SAXS, with structural parameters extracted using a multicomponent model (see Supporting Information for the details of “Modeling SCs scattering”). The scattering pattern observed is consistent with 3D superstructures ordered in a BCC lattice with a constant (*a*_bcc_) of 13.3 ± 0.2 nm, rather than that expected from colloidally stable NCs (Figure 1b,c). We refer to these ordered superstructures as supercrystals (SCs). Throughout this work, we use superlattice to describe the periodic NC lattice and SC to denote the mesoscopic crystalline assemblies composed of that lattice. In addition to the BCC reflections, the scattering patterns contain peaks corresponding to crystalline PbOA_2_ with an interlamellar spacing of ∼ 4.65 nm (Figures 1c and S2), consistent with the limited solubility of PbOA_2_ in HDE near room temperature. The SC purification process removes the crystalline PbOA_2,_ preserving the BCC structure and yielding rhombic dodecahedral SCs (Figures 1d and S3).

**Figure 1:**
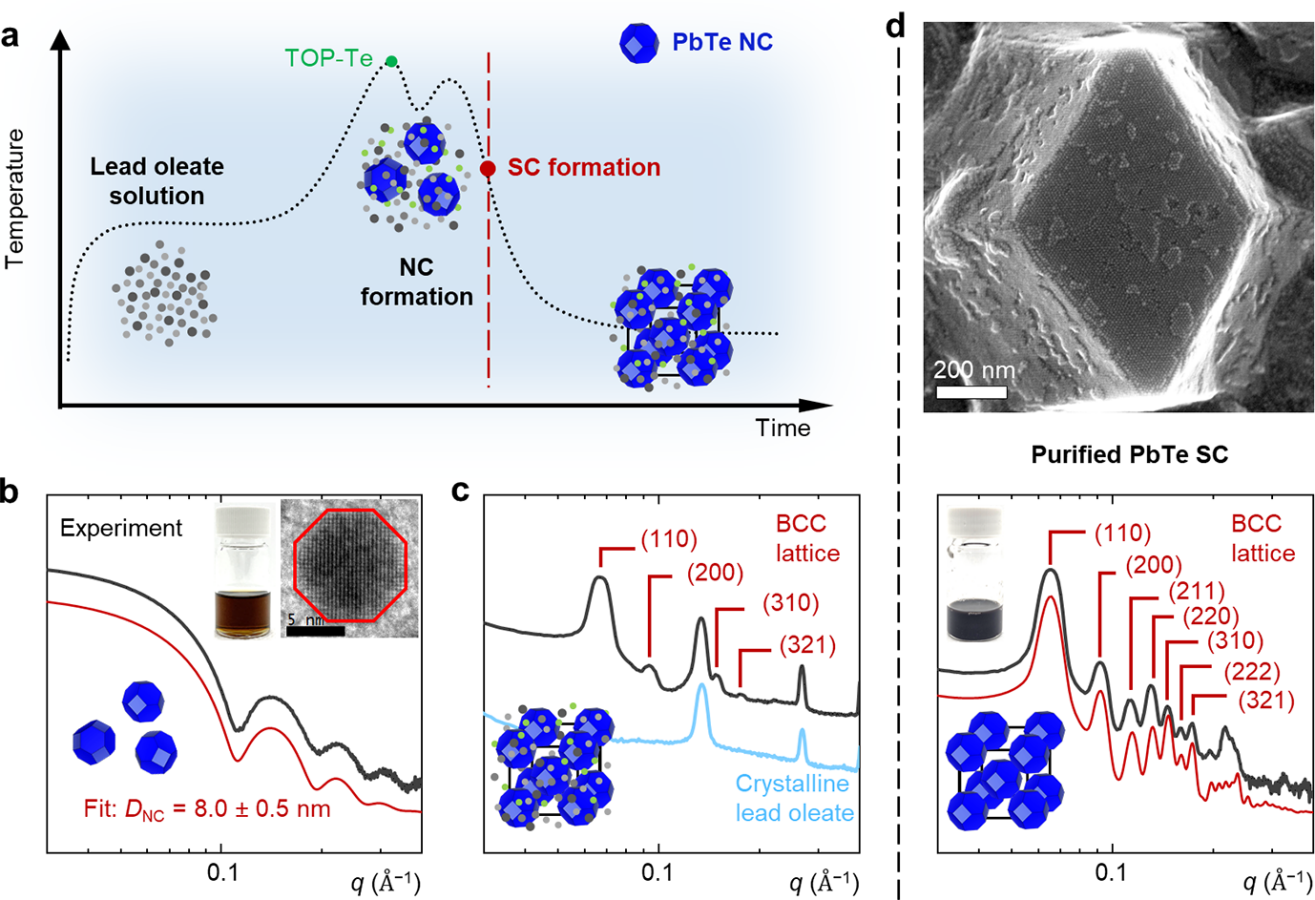
Direct formation of PbTe SCs in the NC reaction medium. (a) Schematic illustration of the synthesis pathway from NC growth to SC formation. Upon cooling, PbTe NCs spontaneously assemble into BCC superlattices within the reaction medium. All SAXS intensities are shown as *I*(*q*) in arbitrary units with logarithmic scaling of *x*- and *y*-axes. (b) Experimental SAXS patterns of colloidally stable PbTe NCs (8.0 ± 0.5 nm) in hexane, fit to a five-parameter sphere model (diameter, polydispersity, surface disorder, volume fraction and background). Insets show a photograph of the PbTe NC solution and a high-resolution transmission electron microscopy (TEM) image of a single NC with a scale bar of 5 nm, revealing a truncated NC shape. (c) SAXS patterns of the crude solution at room temperature after NC synthesis. The peaks arise from a BCC lattice (red) and from crystalline lead oleate present in HDE (blue). (d) Scanning TEM-secondary electron image (top) and SAXS pattern (bottom) of purified SC. The inset shows the SC suspension in toluene, used for the SAXS measurement. The red curve shows the simulated scattering pattern for a BCC lattice, with the fitting parameters described in Table S5.

Previous work demonstrated that assembly during NC synthesis can be driven by van der Waals attraction between NCs once they reach a sufficiently large size to overcome ligand-induced repulsion and form SCs.^26,28,31,33,34^ To evaluate whether this mechanism could account for the assembly in our system, we modeled the pairwise interaction potential between oleate-capped 8 nm PbTe NCs as the sum of van der Waals attraction and steric repulsion from the ligands (Figure 2a). The model yields a shallow potential minimum of −0.1 *k*_B_ *T* at a NC core-to-core distance of 12.0 nm (Figure 2a and see Supporting Information for the details of “Calculation of NC–NC interaction potential”). In comparison, the SAXS data show a shorter experimental spacing of 11.5 ± 0.2 nm (Table S5). The shallow potential depth indicates that van der Waals attraction alone is insufficient to stabilize the observed long-range ordering, suggesting that additional short-range attraction must be present.

**Figure 2:**
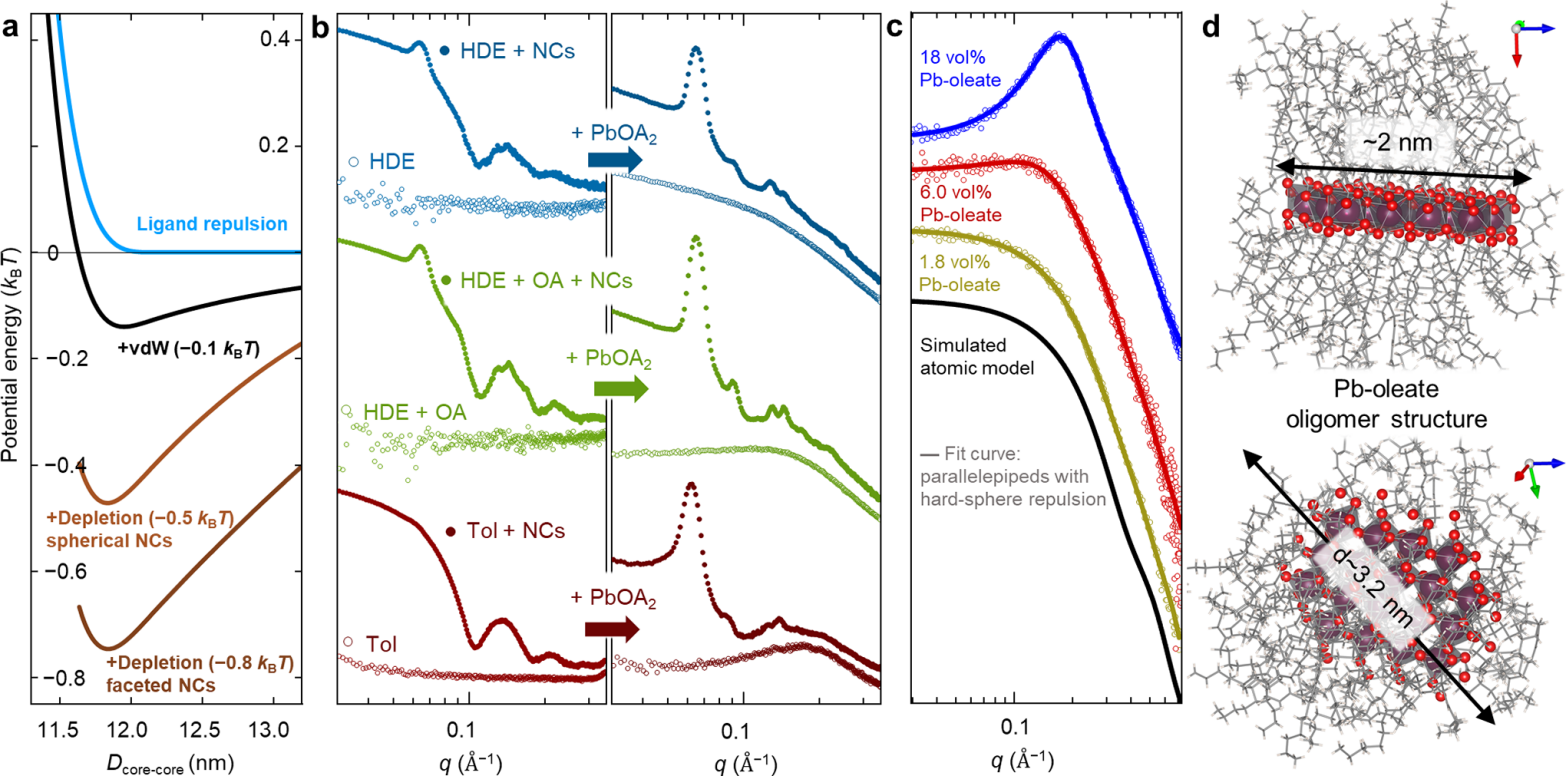
Origin of depletion-driven PbTe SC assembly. All SAXS intensities are plotted as *I*(*q*) in arbitrary units with logarithmic scaling of *x*- and *y*-axes. (a) Calculated NC–NC interaction potentials as a function of NC core-to-core distance for 8 nm PbTe nanocrystals. (b) SAXS patterns of oleate-capped PbTe NCs dispersed in different environments. Left panel shows scattering from toluene (Tol), hexadecane (HDE), and hexadecane with added oleic acid (HDE + OA), measured for both the neat solvents and the corresponding NCs dispersions. Right panel shows the analogous data sets after the addition of PbOA_2_. (c) SAXS patterns and fits for PbOA_2_:OA mixtures in HDE at different concentrations, together with the simulated scattering curve based on a lamellar form factor. (d) Molecular models of Pb**–**oleate oligomers composed of multiple lead oleate units (∼2 nm), corresponding to an effective solvated diameter of ∼3.2 nm in solution.

To identify the source of this attraction, we first tested whether the precursor environment alone is sufficient to induce SC formation. Although SCs appear in the PbTe synthesis medium, this could, in principle, arise from transient processes during the reaction, such as nucleation kinetics or byproducts. To decouple these effects, we reconstructed an “artificial crude solution” containing HDE, PbOA_2_, OA, and TOP-Te at concentrations similar to those present during the synthesis, but without heating or ongoing precursor conversion. Purified NCs added to this mixture at room temperature immediately produced a cloudy suspension, and SAXS confirmed BCC ordering identical to that observed in the synthesis medium (Figure S4). The precursor environment alone, independent of synthesis kinetics, is therefore sufficient to drive assembly.

To identify which species are responsible, we combined well-dispersed NCs with different subsets of the reaction mixture components (Figures 2b and S5). In the absence of PbOA_2_, SAXS patterns display only weak aggregation signatures, along with form-factor oscillations characteristic of well-dispersed NCs. We attribute this weak aggregation to trace amounts of PbOA_2_ that remain associated with the NCs after purification, as confirmed by nuclear magnetic resonance (NMR), which are difficult to fully remove without compromising colloidal stability (Figure S6). In contrast, the presence of PbOA_2_ triggers BCC ordering, and the effect is further enhanced when both PbOA_2_ and OA are combined, resulting in sharper Bragg peaks. Critically, in toluene, where NCs remain well dispersed, no ordering is observed unless PbOA_2_ is added, confirming that assembly is not driven by solvent quality but by PbOA_2_ (Figure 2b). Since van der Waals forces are present in all tested media, this selectivity identifies PbOA_2_-derived species as necessary for assembly and motivates closer examination of their structure in solution.

SAXS measurements of PbOA_2_:OA in HDE show that PbOA_2_ is not present as isolated molecular species but instead forms platelet-like structures with dimensions of ∼2 nm (Figure 2c), well above the molecular length scale expected for monomeric lead oleate complexes.^39^ Modeling the SAXS data using particle form factors corresponding to thin, anisotropic platelets, consistent with the coordination chemistry and structural motifs known for lead oleate in apolar environments,^40,41^ indicates the presence of supramolecular Pb–oleate oligomers composed of multiple Pb–oleate units with variable oleic acid coordination (Figure 2d and see Supporting Information for the details of “Structure of Pb–oleate oligomers in HDE). These supramolecular oligomers persist across the full temperature range relevant for NC synthesis and assembly (30–160 °C), with only a minor reduction in effective diameter upon heating, consistent with temperature-dependent modification of solvation rather than any change in their fundamental oligomeric structure (Supporting Information Note 4 and Figure S15). Aggregation of lead carboxylates into oligomeric or micellar species in apolar media has long been recognized in the colloid chemistry of metal soaps,^41–44^ and oleate oligomers of comparable size have been identified as synthesis byproducts in PbS quantum dot samples by NMR,^42^ consistent with the ∼3.2 nm effective solvated size determined here by SAXS. This effective solvated size places the depletion range on the order of the ligand shell thickness, making it commensurate with the interparticle separation relevant for superlattice formation and explains why depletion strengthens interparticle attraction without compressing the ligand shell, preserving the conditions necessary for reversible ordering rather than irreversible aggregation.

These oligomers satisfy the key criterion for depletion agents: they are present in solution but do not adsorb onto the NC surface.^12–14,37^ Nonadsorbing species generate an osmotic pressure when NCs approach within the oligomer exclusion zone, producing a short-range attractive potential whose strength scales with oligomer concentration. Incorporating this force into the effective pair potential deepens the well from −0.1 to approximately −0.5 *k*_B_ *T*, without significant compression of the ligand shell since the equilibrium spacing shifts only from 12.0 to 11.8 nm (Figure 2a). This value should be regarded as a conservative lower bound obtained for spherical NCs, with faceting increasing the effective attraction to values approaching −0.8 *k*_B_ *T* (Figure 2a). Prior theoretical analyses supported by experimental studies have shown that ordered phases typically assemble when the well depth is on the order of 0.5–1 *k*_B_ *T*;^45–47^ with deeper wells driving irreversible aggregation. The depletion attraction generated by Pb–oleate oligomers therefore falls precisely within the window required for reversible superlattice formation.

The emergence of BCC ordering provides additional mechanistic insight. Truncated octahedral PbTe NCs match the Voronoi cell of a BCC lattice, making BCC packing geometrically favorable. Under ligand-rich conditions where longer range van der Waals interactions dominate, the effective particle shape is partially rounded by the ligand shell, and face-centered cubic (FCC) packing is commonly observed.^23,48^ Depletion, by contrast, introduces a dominant short-range attraction without significantly compressing the ligand shell, allowing the native core geometry to play a more prominent role in determining the lattice symmetry. Computational studies have shown that reduced ligand packing frustration and higher configurational entropy favor BCC superlattices in this regime,^15,16,49^ consistent with the BCC superlattices observed here across all depletion-driven conditions.

A critical test of the depletion assignment is whether oligomer concentration acts as a single predictive parameter for collective order, behavior expected for depletion-driven assembly but incompatible with van der Waals or ligand-mediated mechanisms. We examined this by varying oligomer content in purified NC dispersions under nonreactive conditions. Increasing oligomer concentration drives a clear transition from dispersed NCs to long-range ordered BCC superlattices (Figure 3a,b), while lattice symmetry and parameter remain unchanged throughout. Depletion strength, therefore, controls the stability of a fixed geometric packing rather than restructuring the lattice. At very high concentrations, overly strong attraction leads to kinetic trapping, limiting structural equilibration while preserving BCC symmetry. The concentration window for stable ordering shifts across solvents, reflecting solvent-dependent modulation of steric repulsion and effective depletion strength.

**Figure 3:**
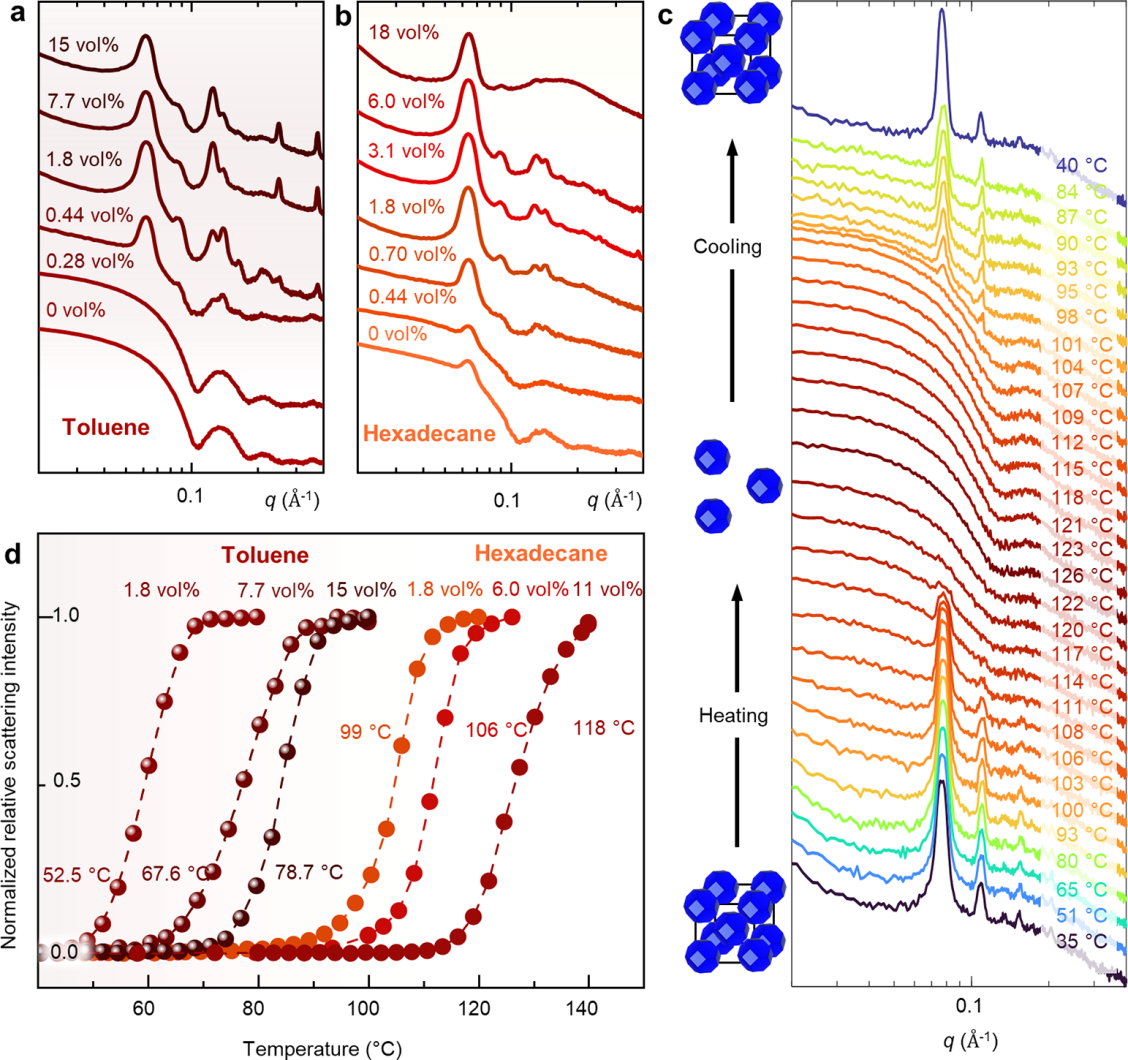
Depletant concentration and temperature enable reversible control over SC formation. All SAXS intensities are plotted as *I*(*q*) in arbitrary units with logarithmic scaling of *x*- and *y*-axes. SAXS patterns showing the emergence of BCC ordering, with different amounts of Pb–oleate oligomers added into toluene (a) and hexadecane (b) NC solutions. (c) Temperature-dependent SAXS demonstrating reversible dissolution and reassembly of PbTe SCs formed with 6.0 vol % Pb–oleate oligomer solution. (d) Normalized ratio of dispersed NC to assembled SC as a function of temperature for different Pb–oleate volume fractions in toluene and hexadecane. A value of 1 corresponds to fully dispersed NCs, whereas 0 indicates complete assembly into SCs. The order–disorder transition temperature (*T*_transition_) is defined by a fixed threshold (see Supporting Information for the experimental and analysis details of “Determination of order–disorder transition temperatures”).

Temperature provides an independent axis for validating the depletion assignment. In an idealized depletion picture, the interaction does not depend explicitly on temperature beyond the thermal energy scale. Temperature, therefore, probes the stability of the ordered phase against thermal fluctuations rather than modifying the interaction itself. This predicts that heating should dissolve the ordered lattice into a dispersed state without altering its symmetry, and that the transition temperature should shift predictably with depletant concentration. Both predictions are confirmed. Upon heating, BCC Bragg reflections progressively weaken and disappear, and cooling fully restores the same BCC ordering (Figure 3c), ruling out kinetic trapping as the origin of order. Increasing the depletant concentration shifts the transition to higher temperatures (Figures 3d and S16–S18), consistent with a stronger effective attraction that requires greater thermal energy to disrupt. The invariance of lattice symmetry across the transition, combined with the predictive dependence of transition temperature on a single chemical variable, establishes depletion-mediated assembly as a controllable thermodynamic process whose order–disorder boundary is set by the composition of the surrounding medium.

Having established that depletion governs superlattice formation in the PbTe model system and confirmed this assignment through predictive control of the order–disorder boundary, we asked whether the same interaction framework could be deliberately activated across chemically and structurally distinct NC systems. If depletion is truly the operative mechanism, introducing depletants of appropriate size and nonadsorbing character into any colloidally stable NC dispersion should be sufficient to induce ordered assembly, independent of NC composition, shape, or synthesis history. We tested this in two distinct contexts: crude reaction mixtures in which spontaneous ordering does not occur under native conditions, and purified NC dispersions with different compositions and geometries.

In crude reaction media, depletion-driven ordering can be deliberately activated in systems that do not assemble spontaneously. PbSe NCs, which remain colloidally stable under their native synthesis conditions, rapidly form BCC supercrystals upon addition of Pb–oleate oligomers during cooling of the reaction medium (Figures 4a and S7a), demonstrating that the absence of spontaneous ordering reflects insufficient depletion strength rather than any fundamental incompatibility between the NCs and ordered assembly. The generality of this activation extends beyond the identity of the depletant itself: Au NCs assemble into FCC superlattices when either Pb–oleate oligomers or polystyrene of comparable size (3.35 kDa) are introduced into crude reaction mixtures (Figures 4b and S7b). The fact that chemically distinct depletants of similar dimensions produce equivalent ordering confirms that depletion is governed by size and nonadsorbing character rather than by specific chemical interactions between the depletant and the NC surface.

**Figure 4:**
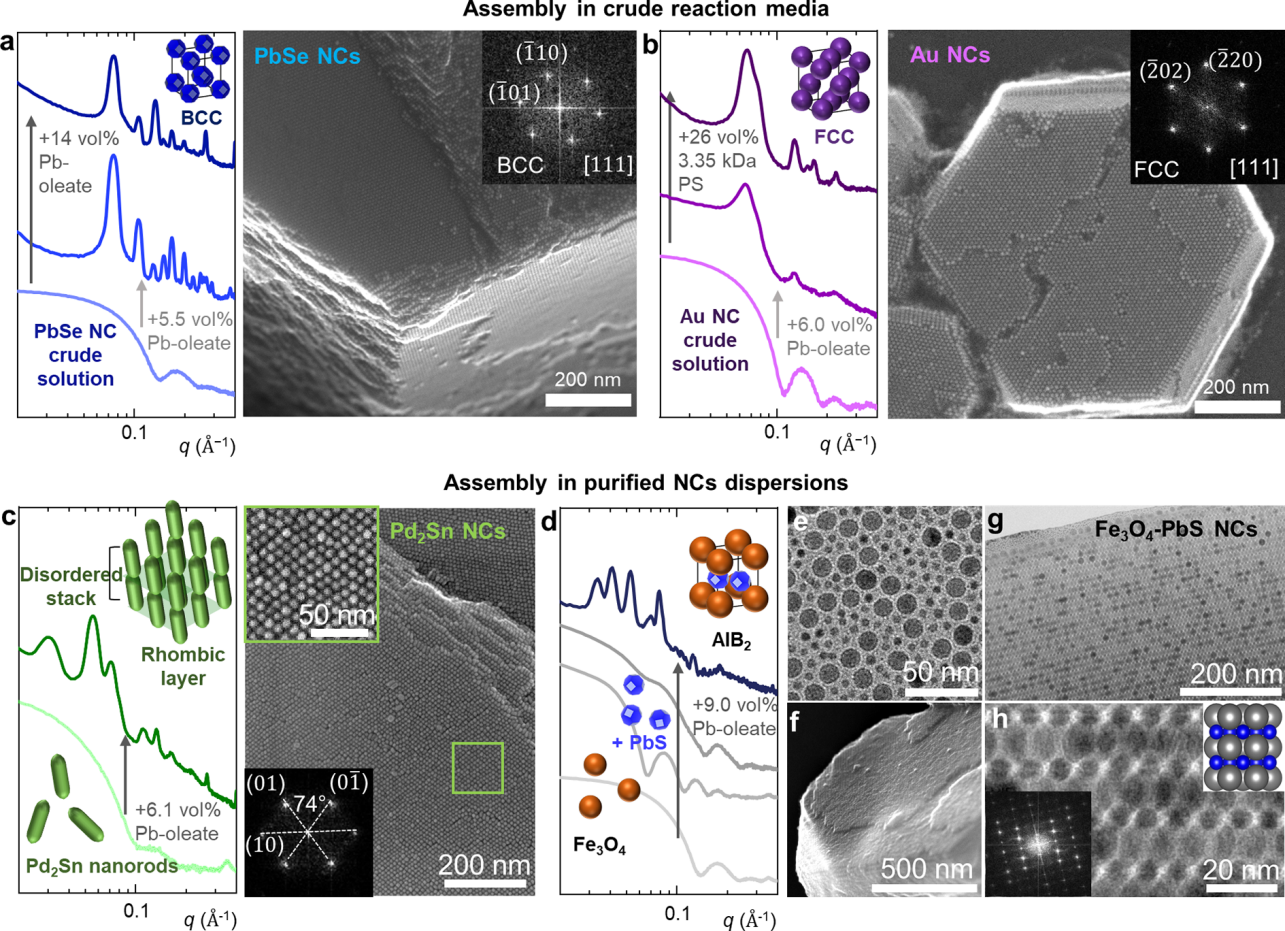
Transferability of depletion-mediated NC superlattice assembly across diverse materials, shapes, and environments. All SAXS intensities are plotted as *I*(*q*) in arbitrary units with logarithmic scaling of *x*- and *y*-axes. (a) Assembly in crude reaction media. SAXS of 6.8 nm PbSe NCs in the reaction mixture showing the formation of BCC-ordered SCs upon addition of Pb–oleate oligomers (5.5 vol % and 14 vol %) at room temperature (left); and a representative scanning TEM-secondary electron image of PbSe SCs, with an inset of a fast Fourier transform (FFT) pattern indexed to the BCC lattice. (b) Assembly in crude reaction media with distinct depletants. SAXS of 8.6 nm Au NCs in the reaction mixture showing the formation of FCC-ordered SCs upon addition of either Pb–oleate oligomers (6.0 vol %) or polymeric depletants (3.35 kDa polystyrene (PS), 26 vol %) at room-temperature (left); and a representative scanning TEM-secondary electron image of Au SCs, with an inset of an FFT pattern indexed to the FCC lattice (right). (c) Assembly of anisotropic Pd_2_Sn nanorods of length 17.3 nm and width 7.4 nm in purified dispersions. SAXS of Pd_2_Sn nanorods in HDE showing in-plane rhombic ordering with disordered stacking out-of-plane after addition of Pb–oleate oligomers (6.1 vol %) at room temperature (left); and a representative scanning TEM-secondary electron image of Pd_2_Sn SCs, with an inset showing a magnified region and the corresponding FFT, revealing a rhombic (oblique) lattice viewed along [001] zone axis with an interplanar angle of 74°. **(d**–**h)** Binary superlattices in purified dispersions. (d) SAXS of 14 nm Fe_3_O_4_ and 6.5 nm PbS NCs in toluene with Pb–oleate oligomers (9.0 vol %), corresponding to an AlB_2_-type hexagonal structure. (e) Representative TEM image of the Fe_3_O_4_ and PbS NCs mixture prior to assembly. (f) Scanning TEM-secondary electron image of a binary SC showing long-range periodic order. **(g**,**h)** Cross-sectional TEM image of a binary SC prepared by cryo-focused ion beam (g), and a magnified region with FFT and structural model consistent with AlB_2_ type ordering (h). Additional structural characterizations of the aforementioned systems are shown in the Figures S7–S9.

The same depletion framework operates in purified dispersions, entirely removed from the synthesis environment. Introducing Pb–oleate oligomers into well-dispersed Pd_2_Sn nanorods yields ordered assemblies characterized by in-plane rhombic symmetry (Figures 4c and S8), showing that depletion can direct the organization of anisotropic particles where shape anisotropy introduces additional geometric constraints on packing. Binary mixtures of Fe_3_O_4_ and PbS NCs form compositionally ordered superlattices consistent with an AlB_2_-type structure upon addition of depletants (Figures 4d–h and S9), demonstrating that depletion can simultaneously drive assembly and preserve compositional order in multicomponent systems.

Across all systems examined, from crude reaction media to purified dispersions, from single-component spherical NCs to anisotropic and binary assemblies, lattice symmetry reflects NC geometry while ordering stability is governed by depletant concentration. The chemical identity of the NCs, the nature of the synthesis environment, and the specific depletant used are secondary to a single controlling variable: the strength of the depletion attraction set by the surrounding medium. This separation of interaction strength from structural and chemical identity is what makes depletion a transferable and rationally controllable framework for collective organization across the broad compositional and geometric space accessible to colloidal NC synthesis.

## Conclusions

The results presented here establish that the chemical environment surrounding NCs during synthesis is not a passive background but an active contributor to collective organization. Precursor-derived oligomeric species, long present in reaction media but not previously identified as interaction-relevant, can generate depletion attractions sufficient to drive ordered superlattice formation. This reframes how assembly in chemically complex environments should be interpreted and, more practically, how it can be designed. Rather than treating the reaction medium as a constraint to work around, its composition can be tuned to program interparticle interactions directly.

Because depletion strength is set by the surrounding medium independently of NC surface chemistry, this strategy is broadly transferable. The same interaction framework activates ordering across compositions, shapes, and multicomponent systems without requiring surface modification or precisely controlled processing conditions. This compositional flexibility, combined with the thermodynamic reversibility demonstrated here, opens a route to superlattice formation that is compatible with the full diversity of colloidal NC synthesis and adaptable to systems where conventional assembly strategies are difficult to apply.

## Supplementary Material



## Notes

Experimental and characterization details (chemicals used, NC and SC synthesis methods, and photographs of SAXS setup); SAXS sample preparation (including the artificial reaction medium, PbOA_2_:OA stock solutions, and purified NC-precursor mixtures); SAXS analysis of NCs, SCs, Pb–oligomer solutions, and order–disorder transition temperatures; NC–NC interaction potential calculations; additional experimental data (TEM/STEM images, SAXS patterns, and NMR spectrum) (PDF)
