# Supplementary material for: Reaction Medium as an Architect of Nanocrystal Superlattices
Source: J Am Chem Soc. 2026 Jul 15;148(29):31245–52. doi: 10.1021/jacs.6c07859 (PMC13426261; doi:10.1021/jacs.6c07859)
Supplement: Supplementary file 1 [file ja6c07859_si_001.pdf]

Supporting Information

Reaction Medium as an Architect of Nanocrystal Superlattices

Seungho Lee[†], Daniel M. Balazs[†], Aiswarya Rayaroth[†], Sharona Horta[†], Carl P. Goodrich[†], Michael Engel[‡], Ihor Cherniukh[†], Maria Ibáñez[†]*

[†]Institute of Science and Technology Austria (ISTA), Klosterneuburg 3400, Austria

[‡]Institute for Multiscale Simulation, Friedrich-Alexander-Universität Erlangen-Nürnberg;, Erlangen, 91058, Germany

* E-mail: mibanez@ist.ac.at

Table of Contents

Material synthesis 3
Chemicals 3
Synthesis of lead oleate ($PbOA_2$) powder 5
Synthesis of 8 nm PbTe nanocrystals (NCs) and supercrystals (SCs) 6
Synthesis of 6.8 nm PbSe NCs 7
Synthesis of 6.5 nm PbS NCs 7
Synthesis of Pd_2Sn nanorod (length: 17.3 nm, width: 7.4 nm) 8
Synthesis of 14 nm Fe_3O_4 NC 9
Synthesis of 8.6 nm Au NC 9
Artificial crude reaction medium preparation 10
$PbOA_2$:OA stock solution preparation 11
Preparation of solvent and precursor mixtures for SAXS 12
Direct SC formation in crude solution (Figures 3a,b) 14
Depletion-derived self-assembly using purified NCs (Figures 3c–h) 14
SAXS characterization 15
SAXS modeling 15
Calculation of NC–NC interaction potential 16
Supporting Figures 19
Supporting Note 1: Form factor of NCs 29
Supporting Note 2: Modeling SCs scattering 32
Supporting Note 3: Structure of Pb–oleate oligomers in HDE 35
Supporting Note 4: Thermal stability of Pb–oleate oligomers under reaction conditions 41
Supporting Note 5: Determination of order-disorder transition temperatures 43
Setup for special SAXS measurements 47
References 49

Material synthesis

Chemicals

Lead oleate ($PbOA_2$) powder synthesis: Lead oxide [PbO, Alfa, 99.9999%], trifluoroacetic anhydride [TFAA, 99%, Thermofisher], trifluoroacetic acid [TFA, 99%, Alfa], oleic acid [OA, 90%, Sigma], acetonitrile [ACN, extra dry, Acros], methanol [extra dry, Acros], and isopropanol [IPA, extra dry, ThermoFisher], triethylamine (99%, ThermoFisher) were purchased. OA was recrystallized using acetone three times before use.

PbTe nanocrystal (NC) and supercrystal (SC) synthesis: hexadecane [HDE, 99%, ThermoFisher], tellurium shot [Te, granule 2-5 mm, 99.999%, ThermoFisher], Tri-n-octylphosphine [TOP, 97%, Strem], OA [90%, Sigma], toluene [anhydrous, Sigma], hexane [anhydrous, Sigma], chloroform [anhydrous, Sigma] were purchased. OA and HDE were degassed at 100 °C for an hour before use.

PbS and PbSe NC: lead oxide [PbO, 99.99%, Alfa], lead acetate trihydrate [$Pb(OAc)_2 \cdot 3H_2O$, Thermofisher, reagent grade], hexamethyldisilathiane [$(TMS)_2S$, synthesis grade, Sigma], 1-octadecene [ODE, 90%, Sigma], diphenylphosphine [DPP, Sigma, 98%], selenium [Se, 99.99% trace metal basis, Sigma], toluene [anhydrous, Sigma], hexane [anhydrous, Sigma], tetrachloroethylene [Fisher, general purpose], and acetone [anhydrous, Acros]. OA and TOP used were purchased from the same supplier with the identical purity as described above. ODE was degassed at 100 °C for an hour before use.

Pd_2Sn nanorod: methylamine hydrochloric acid [MHA, 99%, ThermoFisher], palladium acetylacetonate [$Pd(acac)_2$, 99%, Sigma], tin acetate [$Sn(OAc)_2$, Sigma], oleylamine [OLAM, 80–

90%, ThermoFisher], toluene [anhydrous, Sigma]. OA and TOP used were purchased from the same supplier with the identical purity as described above. OLAM was distilled before use.

Fe_3O_4 NC: Iron chloride hexahydrate [$FeCl_3 \cdot 6H_2O$, 98%, Sigma], sodium oleate [NaOA, 97%, TCI], and 1-octadecene [ODE, 90%, Sigma], OA [90%, Sigma], ethanol [98%, Honeywell], acetone [anhydrous, Acros], hexane [general grade, Sigma].

Au NC: Gold chloride trihydrate [$HAuCl_4 \cdot 3H_2O$, 99.9%, Sigma], tert-butylamine-borane complex [TBAB, 97%, Sigma], hexane [general grade, Sigma], ethanol [98%, Honeywell], toluene [anhydrous, Sigma], acetone [anhydrous, Acros], and OLAM [80–90%, ThermoFisher]. OLAM was distilled before use, otherwise chemicals were used as received.

Synthesis of lead oleate ($PbOA_2$) powder

Crystallized $PbOA_2$ powder was synthesized by adapting the previously reported procedure[1]. In brief, PbO (3.95 g) and ACN (8 mL) were added to a 100 mL round-bottom flask. The suspension was stirred while being cooled in an ice bath (0 °C) for 10 min, after which TFA (0.3 mL) and TFAA (2.5 mL) were sequentially introduced into the flask. After 1 h at 0 °C, lead oxide got dissolved (forming yellowish cloudy solution with no visible powders) and the mixture was allowed to warm up to room temperature for 2 h, resulting in a clear and colorless solution. Meanwhile, OA (11.2 mL, 10 g, recrystallized), IPA (75 mL), and triethylamine (5.5 mL) were added to another 100 mL 3-necked round-bottom flask. The TFA solution was then dropwise added to the OA solution with vigorous stirring, resulting in the immediate formation of white precipitates. Following complete addition, the mixture was heated to reflux (75–80 °C) by using an oil bath until the precipitate dissolved into a clear, colorless solution. Upon dissolution, the heating was stopped and the flask was allowed to slowly cool to room temperature over 2 h, followed by further cooling in a −20 °C freezer for 2 hours. The white powder was collected by vacuum filtration using a Buchner with a paper filter and was thoroughly washed with 50 mL of cold methanol 4 times. During purification, the slurry was stirred and any large pieces were broken up. The resulting powders were dried under vacuum (< 0.1 mbar) overnight. The fluffy white powder was recrystallized twice with hot IPA (75 °C) under reflux. Note that the minimum IPA was added until the powder was just immersed. Following the filtration and drying, the white powder was stored in a nitrogen-filled glovebox. The yield was about 90% (~12 g).

Synthesis of 8 nm PbTe nanocrystals (NCs) and supercrystals (SCs)

PbTe NCs were prepared by modifying the reported method[2]. In a typical synthesis, $PbOA_2$ powder (0.8536 g, 1.1 mmol) was mixed with degassed OA (0.4 mL) and degassed HDE (7.2 mL) in a 50 mL three-necked round-bottom flask inside the glove box. The reaction flask was connected to the Schlenk line, the mixture was degassed at room-temperature under vacuum (<150 mTorr), and then heated to 80 °C and maintained at this temperature for 30 min. The resulting colorless solution was gradually heated to 160 °C under argon flow and maintained at this temperature for 5 min to attain thermal equilibrium. Subsequently, 1 mL of 1 M TOP-Te precursor was rapidly injected, and the reaction was allowed to proceed for 3 min. The clear brown reaction mixture was then quenched by immersion in a water bath. Upon cooling, the solution became turbid black near 100 °C (Figure S1), at which point the flask was transferred to the glove box for purification. The crude mixture was centrifuged at 5000 rpm for 1 min, the pale yellow supernatant was discarded, and the precipitate was redissolved in 2 mL of $CHCl_3$. The resulting clear brown solution was centrifuged again at 5000 rpm for 3 min to remove residual impurities. The brown supernatant was then mixed with 0.3 mL of anhydrous ACN and centrifuged at 5000 rpm for 3 min. The collected NCs were redispersed in anhydrous apolar solvents such as hexane, cyclohexane, or chloroform. These solvents were selected to ensure good NC dispersibility and to provide higher volatility for control experiments, compared with toluene. The typical yield per batch was ~50 mg of dried nanocrystals. For isolating PbTe SCs from the reaction mixture, the quenched black crude solution was heated up to 80 °C with a ramp rate of 2.7 °C min^{-1} under an argon blanket and then maintained at this temperature for 30 min. For purification, the mixture was centrifuged at 5000 rpm for 1 min, and the pale yellow supernatant was removed. The resulting black precipitate was redispersed in 5 mL of toluene and centrifuged at 4000 rpm for 3 min. After discarding the clear supernatant,

the precipitate was washed once more with the same volume of toluene under identical centrifugation conditions. The purified SCs were finally redispersed in anhydrous toluene for further characterization.

Synthesis of 6.8 nm PbSe NCs

PbSe NCs were synthesized using the following method[3]. In a 100 mL round-bottom three neck flask, PbO (0.892 g, 4 mmol) and OA (3.2 mL, 10 mmol) were mixed in ODE (15.5 mL) to yield a precursor solution. This solution was then degassed by heating stepwise (20 °C every 10–15 minutes) to 110°C over the course of 1 h under vacuum, after which the flask was filled with argon, and the solution was heated to 160 °C. In a glovebox, Se was dissolved in TOP to yield a 1.67 M stock solution, and DPP was added to the TOP-Se solution at a final concentration of 0.1 M. Next, 7 mL of the prepared TOP/DPP-Se solution was rapidly injected into the vigorously stirred, hot lead oleate solution. PbSe NCs formed immediately after injection, the temperature dropped to about 145 °C, and the crystal growth was allowed to proceed for 3 min. The reaction was quenched by removing the heating mantle (~2 min 45 s), injecting 5 mL of toluene (in ~10 s) and placing the flask in a water bath. Following the synthesis, the NCs were washed 2–3 times by sequential precipitation with acetone (2–2.5:1 ratio) and redispersion in hexane.

Synthesis of 6.5 nm PbS NCs

PbS NCs were synthesized following the reported procedure[4]. Specifically, $Pb(OAc)_2 \cdot 3H_2O$ (0.379 g, 1 mmol) was dissolved in OA (7 mL, 22.2 mmol) and ODE (3 mL) in a 25 mL round-bottom three-neck flask and degassed by gradual heating to 105 °C under vacuum for 1 h. The solution was then heated to 146 °C under an argon atmosphere, after which 105 μL of TMS_2S (0.5 mmol) dissolved in 5 mL of ODE was injected into the reaction mixture. The heat source was

immediately removed, and the reaction continued for 3 min before quenching by immersing the flask in an ice-water bath and injecting 10 mL of hexane. The NCs were isolated by the addition of 10 mL of ethanol, followed by centrifugation at 7800 rpm for 2 min. The precipitate was dissolved in 5 mL of hexane and washed twice by adding 5 mL of acetone and centrifuging at 7800 rpm for 2 min. After the second centrifugation, the NCs were dispersed in 1.2 mL of hexane. The dispersion was additionally centrifuged at 7800 rpm for 2 min to remove any bulk aggregates or large NCs. The NC concentration (137 μM) was determined gravimetrically, accounting for a ligand mass fraction of 22% of the total NC mass as determined by thermogravimetric analysis.

Synthesis of Pd_2Sn nanorod (length: 17.3 nm, width: 7.4 nm)

Pd_2Sn nanorods were synthesized by modifying the reported method[5]. In a 25 mL three-neck round-bottom flask, distilled OLAM (5 mL), MHA (15.30 mg, 0.215 mmol), $Pd(acac)_2$ (22.85 mg, 0.075 mmol), and $Sn(OAc)_2$ (8.9 mg, 0.0376 mmol) were mixed by stirring. After degassing this mixture at room temperature under vacuum, the orange solution was heated to 60 °C under argon flow and held for 30 min. Then, 0.25 mL of TOP was added, and the mixture was heated to 200 °C with a ramp rate of 12 $°C\cdot min^{-1}$. The dark reaction mixture was maintained at 200 °C for 30 min, then heated to 300 °C at a heating rate of 2.5 $°C\cdot min^{-1}$. After being held at 300 °C for 15 min, the solution was cooled by blowing air. The crude Pd_2Sn nanorods were first purified by adding 250 μL of degassed OA, followed by centrifugation at 3000 rpm for 5 min. The resulting precipitate was redispersed in 2 mL of toluene with 500 μL of acetone and centrifuged again at 3000 rpm for 3 min. The final precipitate was redispersed in toluene for further use.

Synthesis of 14 nm Fe_3O_4 NC

Iron oxide NCs were synthesized using the reported method[6]. For the synthesis of the iron oleate complex, $FeCl_3 \cdot H_2O$ (2.705 g, 10 mmol) and sodium oleate (9.125 g, 30 mmol) were dissolved in a mixture of hexane (35 mL), ethanol (20 mL), and distilled water (15 mL). After stirring under nitrogen at 60 °C for 5 h, the upper organic layer was separated and washed four times with a mixture of warm water (30 mL) and ethanol (4 mL), and once with warm water (35 mL), each time followed by centrifugation and decantation of the upper layer. The waxy solid product was obtained by vacuum drying at 50 °C and then dissolved in ODE to form a 0.4 mol/kg solution. For the synthesis of NCs, iron oleate complex in ODE (4 mL), OA (0.64 mL, 2 mmol), and ODE (4 mL) were loaded into a 25 mL three-neck flask and vacuum-dried at 120 °C for 50 min. The reaction mixture was then heated under argon to 312 °C in 17 min and stirred at this temperature for additional 36 min. The flask was then cooled to room temperature with a stream of compressed air. The nanoparticles were isolated by adding hexane (8 mL) and acetone (8 mL), followed by centrifugation at 8000 rpm for 3 min. The precipitate was dissolved in hexane (3.2 mL) with OA (40 μL). After three additional rounds of purification with acetone (2.4 mL, 2.4 mL, 1.6 mL, respectively) the NCs were dispersed in 1.6 mL of hexane. The dispersion was additionally centrifuged at 11000 rpm for 2 min to remove any bulk aggregates or large NCs. The NC concentration (5.1 μM) was determined gravimetrically, accounting for a ligand mass fraction of 13% of the total NC mass as determined by thermogravimetric analysis (using Mettler Toledo TGA/DSC 3+).

Synthesis of 8.6 nm Au NC

To synthesize 8.6 nm Au NCs, 5.9 nm Au NC seeds were first prepared following the reported procedure[7]. In a 25 mL three-neck flask, $HAuCl_4 \cdot 3H_2O$ (49.3 mg, 0.125 mmol) was dissolved in

a mixture of 5 mL hexane and 5 mL OLAM. The solution was bubbled with argon for 15 min. Next, TBAB (21.75 mg, 0.25 mmol), pre-dispersed by vortexing in a mixture of 0.5 mL of hexane and 0.5 mL of OLAM, was then rapidly injected at room temperature. The solution turned deep purple within 5 s, and stirring was continued for 40 min. The Au seeds were purified by adding 14 mL of acetone followed by centrifugation at 8000 rpm for 2 min. The precipitate was redispersed in toluene (2 mL), washed again by addition of acetone (6.1 mL) and ethanol (2.3 mL), and centrifuged at 8000 rpm for 1 min. Finally, the Au seeds were redispersed in 2 mL of toluene.

Au NCs with an average diameter of 8.6 nm were synthesized via seeded growth by reduction of $HAuCl_4 \cdot 3H_2O$ in OLAM using the 5.9 nm Au seeds[8]. In a 25 mL three-neck flask, $HAuCl_4 \cdot 3H_2O$ (51 mg, 0.129 mmol) was dissolved in 2.7 mL of toluene and 2.5 mL of OLAM, followed by addition of 1.3 mL of the Au seed solution The reaction mixture was stirred under argon at 90 °C for 4 h and then allowed to cool to room temperature. The crude solution was stored for further use.

Artificial crude reaction medium preparation

To reconstruct the post-synthesis reaction medium, we estimated the NC reaction yield to determine the PbTe NC concentration and the amount of unreacted precursors remaining in solution. Reaction byproducts were not considered. The total NC material recovered after purification was weighed, and the inorganic PbTe mass was obtained by correcting the dry mass for the organic fraction determined by thermogravimetric analysis. The organic fraction was independently assessed by quantifying oleate species by solution nuclear magnetic resonance (NMR) spectroscopy, allowing estimation of the ligand mass associated with the purified NCs. In parallel, the NC concentration was determined from UV-Vis-NIR absorption spectroscopy using

the NC extinction coefficient. Based on the consistency of these approaches across batches, a representative NC yield of approximately 20% was adopted for reconstruction of the reaction medium and all control experiments. To convert inorganic PbTe mass into NC number concentration, the number of PbTe formula units per NC was estimated from the mean particle diameter obtained by TEM, assuming spherical geometry and bulk stoichiometry.

Based on this representative yield, an artificial crude solution was prepared by mixing the estimated amounts of unreacted $PbOA_2$ and OA in HDE and heating the mixture to 80 °C to ensure $PbOA_2$ solubility. After cooling to 40 °C, TOP-Te and PbTe NC were added at the concentrations expected in the reaction medium.

$PbOA_2$:OA stock solution preparation

$PbOA_2$ stock solutions in HDE containing OA were prepared at different nominal lead oleate concentrations (1.8, 6.0, 8.8, 12, 18, 55 vol%) by mixing $PbOA_2$ powder with OA and HDE at room temperature. The lead oleate oligomer concentration was expressed as volume fraction and was calculated by dividing the volume of added $PbOA_2$ (considering its density of 1.4 g cm^{-3}) by the total volume of solutions, assuming volume additivity and ideal mixing. The amount of OA added was such that the molar ratio of $PbOA_2$:OA was kept constant 1:1.4. To dissolve the $PbOA_2$, the mixture was heated up to 80 °C. Between 40–50 °C, the solution becomes transparent. Once the solution reached 80 °C, it was kept at that temperature for 30 min, after which it was cooled down. In order to avoid the precipitation of $PbOA_2$, the solution was kept between 40–45 °C.

Preparation of solvent and precursor mixtures for SAXS

All sample preparations were carried out inside the N_2-filled glove box. Unless stated otherwise, NCs were purified as described prior to controlled mixing with oligomeric depletants and subsequent assembly experiments. SAXS samples corresponding to each main figure and supporting figure were prepared following the protocols and compositions reported below. Throughout this work, the concentration of added depletants was expressed as a volume fraction. Volume additivity and approximately ideal mixing were assumed. The resulting uncertainty in the estimated volume fraction is estimated within 10%. NC dispersions were prepared by adding PbTe NCs to the desired medium. After NC addition, all samples were placed under vacuum for 10 min to remove the NC carrier solvent (hexane or chloroform). Samples were then stirred, loaded into glass capillaries, and sealed for SAXS measurements. Samples containing Pb–oleate were measured at *ca.* 40 °C using a temperature-controlled sample holder to avoid $PbOA_2$ precipitation.

Table S1. Amounts of precursors, solvent, and NCs used to prepare control mixtures for small-angle X-ray scattering (SAXS) measurements (Figure 2b). Masses are reported for solids and volumes for liquids as used experimentally. For toluene-based samples, HDE was replaced by an equal volume of toluene. NC mass refers to the mass of dried purified NCs.

<table>
<tr><th>Sample</th><th>$PbOA_2$ (mg)</th><th>OA (μL)</th><th>HDE (μL)</th><th>1 M TOP-Te (μL)</th><th>8.5 nm NCs (mg)</th></tr>
<tr><td>HDE+NC</td><td>-</td><td>-</td><td rowspan="6">300</td><td>-</td><td rowspan="6">~2.9</td></tr>
<tr><td>HDE+OA+NC</td><td>-</td><td>16.5</td><td>-</td></tr>
<tr><td>HDE+TOP-Te+NC</td><td>-</td><td>-</td><td>33</td></tr>
<tr><td>HDE+$PbOA_2$+NC</td><td>28.5</td><td>-</td><td>-</td></tr>
<tr><td>HDE+$PbOA_2$+OA+NC</td><td>28.5</td><td>16.5</td><td>-</td></tr>
<tr><td>TOL+$PbOA_2$+OA+NC</td><td>28.5</td><td>16.5</td><td>-</td></tr>
</table>

Table S2. Amounts of precursors, toluene, and NCs used to prepare mixtures with varying Pb-oleate oligomer concentration for SAXS measurements (Figure 3a). Masses are reported for solids and volumes for liquids as used experimentally.

<table>
<tr><th>Sample</th><th>55 vol% $PbOA_2$:OA stock solution (μL)</th><th>Toluene (μL)</th><th>9 nm PbTe NCs (mg)</th></tr>
<tr><td>15 vol%</td><td>27</td><td>73</td><td rowspan="5">~0.8</td></tr>
<tr><td>7.7 vol%</td><td>14</td><td>86</td></tr>
<tr><td>1.8 vol%</td><td>3.2</td><td>97</td></tr>
<tr><td>0.44 vol%</td><td>0.8</td><td>99</td></tr>
<tr><td>0.28 vol%</td><td>0.5</td><td>100</td></tr>
</table>

Table S3. Amounts of precursors, toluene, and NCs used to prepare mixtures with varying Pb-oleate oligomer concentration for SAXS measurements (Figure 3b). Masses are reported for solids and volumes for liquids as used experimentally.

<table>
<tr><th>Sample</th><th>8.8 vol% $PbOA_2$:OA stock solution (μL)</th><th>55 vol% $PbOA_2$:OA stock solution (μL)</th><th>HDE (μL)</th><th>8.5 nm PbTe NCs (mg)</th></tr>
<tr><td>18 vol%</td><td>-</td><td>145</td><td rowspan="4">300</td><td rowspan="4">~2.9</td></tr>
<tr><td>6.0 vol% (standard)</td><td>-</td><td>37</td></tr>
<tr><td>3.1 vol%</td><td>-</td><td>18</td></tr>
<tr><td>1.8 vol%</td><td>-</td><td>10</td></tr>
<tr><td>0.70 vol%</td><td>8</td><td>-</td><td>92</td><td>~0.9</td></tr>
<tr><td>0.44 vol%</td><td>5</td><td>-</td><td>95</td><td>~0.9</td></tr>
</table>

Samples for temperature-dependent SAXS measurements **(Figures 3c,d and S16–S18)** were prepared analogously to those used for room-temperature SAXS **(Tables S2,S3)**, using 0.6 mg of 6.3 nm PbTe and 150 μL of oligomer-solvent mixtures.

Direct SC formation in crude solution (Figures 4a,b)

For PbSe NCs, aliquots of Pb–oleate oligomer stock solution were added directly to the PbSe crude reaction medium to access different oligomer concentrations. The same procedure was applied to Au NCs; in this case, both oligomeric stock solution, or, in separate experiments, polystyrene (3.35 kDa) dissolved in toluene was introduced as the depletant.

Depletion-derived self-assembly using purified NCs (Figures 4c–h)

Purified Pd_2Sn nanorods were dispersed in toluene and combined with a $PbOA_2$:OA stock solution to induce depletion-driven assembly. Binary superlattices were prepared by mixing purified Fe_3O_4 and PbS NCs at a defined particle number ratio (PbS:Fe_3O_4, 6.7:1), followed by the addition of the same oligomer depletant.

SAXS characterization

SAXS data were collected on a XEUSS 3.0 laboratory beamline (Xenocs SA). Copper K_α radiation was generated by a microfocus source and point collimated with a 3D Montel mirror. The beam was shaped by two sets of scatterless slits, and direct and scattered beams were detected simultaneously using an Eiger2 1M-pixel array detector (Dectris AG). The beam path was kept under vacuum (< 0.01 mbar), except for a ~5 cm section sealed by Kapton windows where the samples were measured under ambient atmosphere. The samples were loaded in thin-walled 1 mm borosilicate capillaries (WJM-Glas Müller GmbH) and sealed under an inert atmosphere. Scattering from a solvent-filled capillary was collected and subtracted as background for quantitative SAXS analysis. Scattering patterns were collected using slits widths of 0.5 mm or 0.25 mm, a 285 mm sample-to-detector distance, and an exposure time of 150 s. Temperature-dependent SAXS measurements were performed using a custom-made multicapillary heater stage (**Figure S19**), a Linkam temperature stage (HFSX350) (**Figure S20** and see section "**setup for special SAXS measurement"**). Data were accumulated, corrected, and integrated using the XSACT suite (Xenocs SA). Simple data fitting was performed in SaSView [9].

SAXS modeling

SAXS modeling was performed on three sample classes. Purified PbTe NCs dispersed in hexane were analyzed using an isotropic sphere form factor with a lognormal size distribution, and no interparticle correlations were assumed in the accessed q range (Supporting Note 1). Separately, $PbOA_2$ solutions containing OA were analyzed to estimate the characteristic size and geometry of the dispersed Pb–oleate species, using standard form factor models, and at higher concentrations, including interparticle correlations (Supporting Note 3). Samples containing PbTe SCs were then

modeled as a linear superposition of distinct contributions from free (dispersed) NCs, the SCs, crystalline and/or dissolved $PbOA_2$ species. Additional power-law and constant background terms were included, as required to account for low-q scattering and residual background contributions not captured by the particle-based models ("Modeling SCs scattering"). In all cases, oleate ligands were assumed to be contrast-matched to the solvent and were therefore included only through excluded volume effects. Multicomponent fitting was performed using a custom MATLAB based framework. Detailed component models and fitting procedures are described in the Supporting Information, with representative fits and parameters shown in **Figures S10–S15 and Tables S4–S7**

Calculation of NC–NC interaction potential

The interaction potential between PbTe NCs was estimated by summing the van der Waals attraction of the NC cores, the steric repulsion of the ligand shells, and considering the depletion attraction induced by the oligomers. The van der Waals attraction was calculated for two identical spherical, 8 nm PbTe NCs dispersed in a hydrocarbon matrix using: $\frac{U}{kT} = -\frac{A_{131}}{12kT}\left\{\frac{D^2}{d^2-D^2} + \frac{D^2}{d^2} + 2\ln\left(1-\frac{D^2}{d^2}\right)\right\}$, where A_{131} is the non-retarded Hamaker constant, D is the NC diameter, and d is the center to center distance between NC. The symmetric Hamaker constant across a matrix (A_{131}) was calculated based on the Dzyaloshinskii Lifshitz Pitaevskii (DLP) approach[10]. The critical point in this approach is a realistic description of the complex dielectric spectrum. For PbTe, the spectrum was modeled following Adachi's approach[11], with additional harmonic oscillators in the microwave region using established parameters[12], and empirical corrections were applied using tabulated UV and X-ray data[13]. The dielectric function of the hydrocarbon solvent-ligand mixture was approximated using a single oscillator model following Ninham and Parsegian[14]. Calculations

up to $s = 5$ and $m^*\xi_m$ up to 2 keV yielded a non-retarded Hamaker constant of ~0.79 eV in the entire temperature range, corresponding to ~30 k_BT at room temperature or ~25 k_BT at 100 °C (the latter value was used for all the interaction calculations). The uncertainty is in the range of 0.1 eV or ~10%, stemming mostly from the limited accuracy of the far UV range, and does not affect the qualitative conclusions.

Steric repulsion was calculated using the model developed for short dense polymer brushes[15]. We assume a rigid segment length $b = 0.45$ nm (consistent with coarse-grain molecular dynamics parameters for alkyl chains), and effective contour length $L = 5b = 2.25$ nm and ligand grafting density $\Gamma = 4$ OA/nm^2. The interaction potential is given by: $\frac{U}{kT} = \frac{\pi^3}{12}\Gamma^2 L^2 bD\left[-\ln(u) - \frac{9}{5}(1-u) + \frac{1}{3}(1-u^3) - \frac{1}{30}(1-u^6)\right]$ with $u = (d - D)/2h_0$, where $D = 8$ nm is twice the surface radius of curvature and $h_0 = Lb^{2/3}\Gamma^{1/3} = 2.1\ nm$ is the equilibrium brush thickness. The sum of van der Waals attraction and steric repulsion results in a shallow potential well of approximately –0.1 k_BT at d ~ 12 nm. We define an effective hard sphere diameter (D_{HS} ~ 11.6 nm), corresponding to the separation distance at which the total interaction equals zero. This distance is used as the reference contact distance in the depletion model. This choice reflects the fact that ligand shells are expected to undergo partial compression under the osmotic pressure of the depletants, but not to the extent of entering the regime of steep steric repulsion. The depletion interaction between spherical NCs was calculated using an Asakura Oosawa type model with a depletant hard sphere radius $\sigma = 3.2$ nm, obtained from fitting the hard sphere repulsion as described in the "Structure of Pb–oleate oligomers in HDE" section. The depletant volume fraction (ϕ) based on the nominal volume fraction of $PbOA_2$ correspond to $\phi = 0.06$. The depletion potential is given by: $\frac{U}{kT} = -\frac{\phi}{2\sigma^3}[2(D_{HS} + \sigma)^3 - 3(D_{HS} + \sigma)^2 d + d^3]$. This expression applies only when

the exclusion volumes of the two NCs overlap, that is for $D_{HS} \leq d \leq D_{HS} + \sigma$. For larger separations, the depletion interaction is zero. The potential well deepens to –0.5 $k_B T$ at d ~ 11.8 nm, in agreement with the values extracted from SAXS experiments. This spherical model provides a conservative lower bound for the depletion attraction. An upper bound was estimated by calculating the depletion interaction between two flat facets: $\frac{U}{kT} = -\frac{6\Phi}{\pi\sigma^3} A_{\mathrm{facet}}(D_{\mathrm{HS}} + \sigma - d)$. The following assumption were made: 1) the van der Waals and repulsive interactions remain the same (D and D_{HS} unchanged), 2) the particle is a truncated octahedra with D_{HS} distance between the hexagonal facets, 3) A_{facet} is the area of such a hexagonal facet. The potential well is –0.8 $k_B T$ at 11.8 nm in this case. At the ligand-controlled separation relevant here (surface-to-surface gap of several nanometers), the absolute magnitude of the van der Waals attraction is small, and faceting would increase it only by a few hundredths of $k_B T$, which does not alter the resulting upper bound.

Supporting Figures

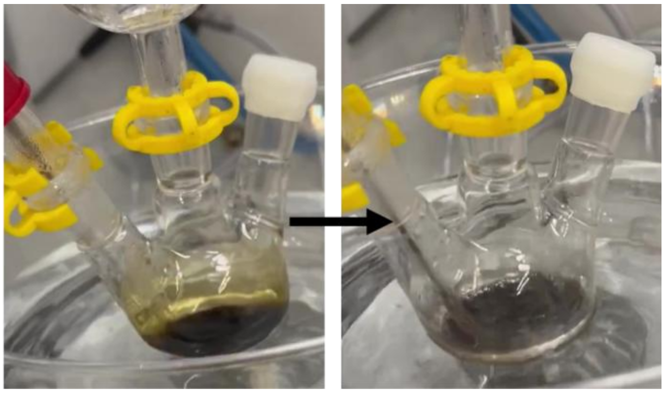

Figure S1. Photographs of the PbTe NC reaction flask after the NCs growth was finished (left) and once the crude solution was cooled down to room temperature (right). Upon cooling, around *ca.* 100 ºC, the solution transitions from a clear yellow-brown appearance to a turbid black dispersion, indicating the onset of strong light scattering associated with NC assembly.

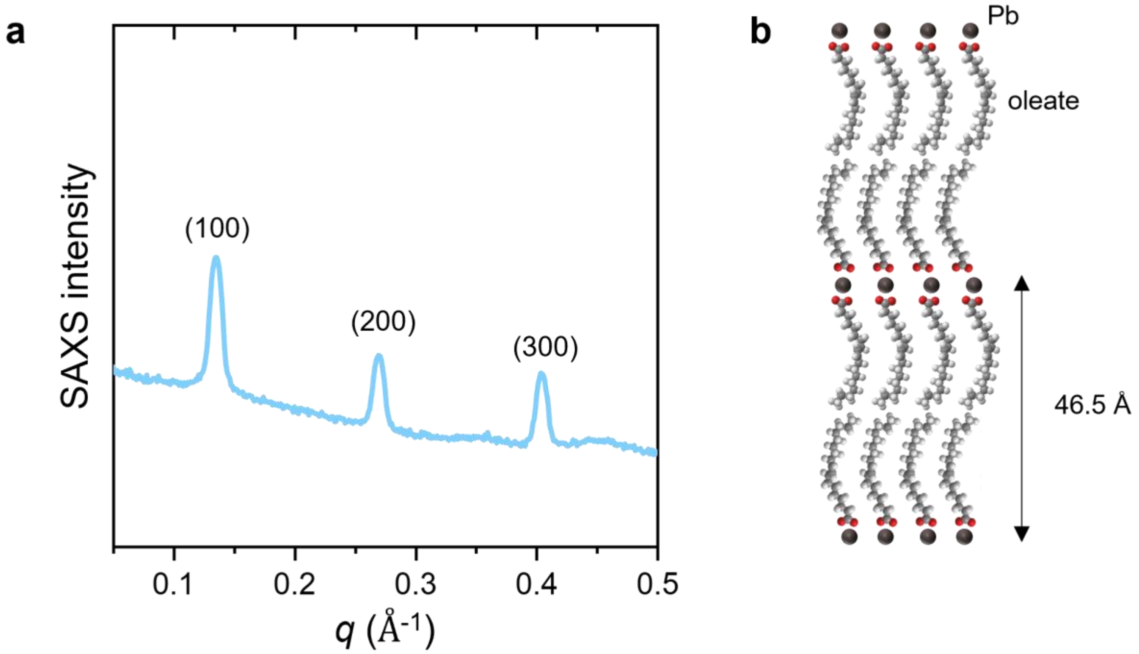

Figure S2. (**a)** SAXS pattern of lead oleate after precipitation from HDE upon cooling to room temperature. At elevated temperature, $PbOA_2$ is fully dissolved, whereas cooling induces phase separation and the formation of a condensed lamellar mesophase. The observed harmonic series of reflections with *q* position ratios of 1:2:3 is characteristic of one-dimensional lamellar ordering. The corresponding repeat distance extracted from the primary peak is *d* = 46.5 Å. (**b)** Conceptual schematic illustrating the lamellar stacking of $PbOA_2$ aggregates within the precipitated phase.

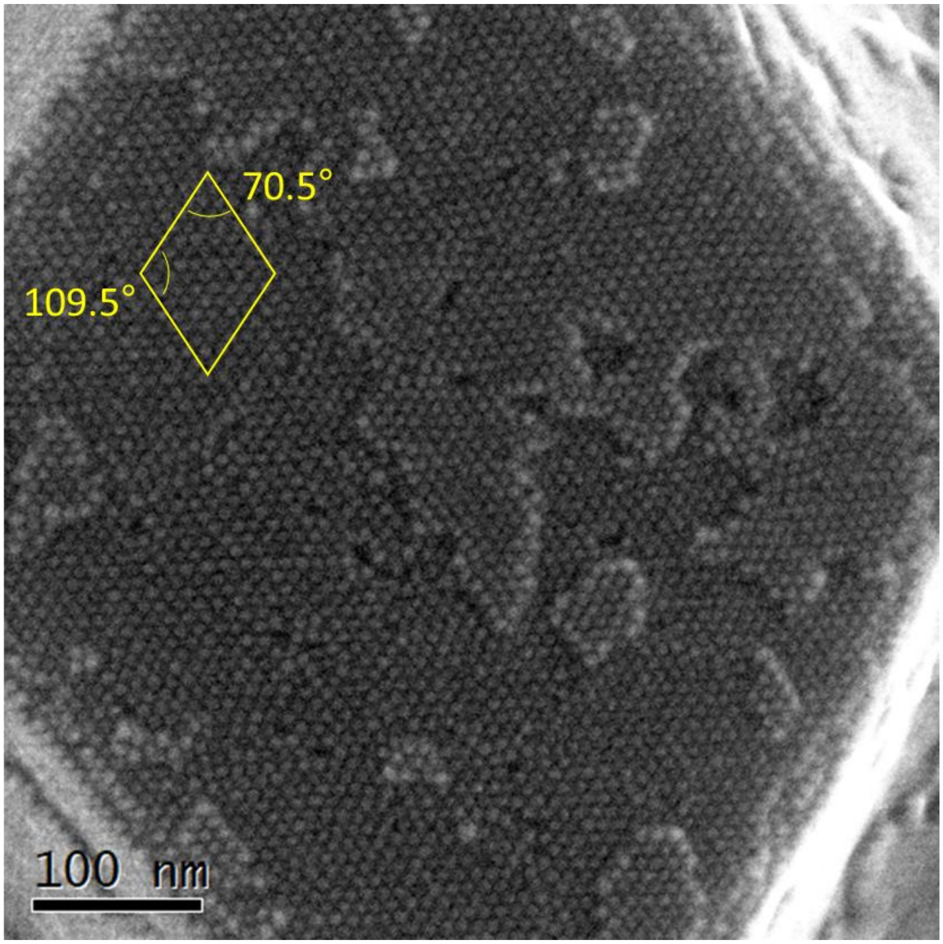

Figure S3. Scanning transmission electron microscopy (STEM) secondary electron (SE) image of a body-centered cubic (BCC) NC superlattice viewed along the [110] zone axis. The projected angles between the $\{1\bar{1}2\}$ and $\{\bar{1}12\}$ lattice directions are highlighted and measured to be 70.5° and 109.5°, consistent with the expected dihedral angles for a BCC lattice. Angles were extracted from the image using *ImageJ*.

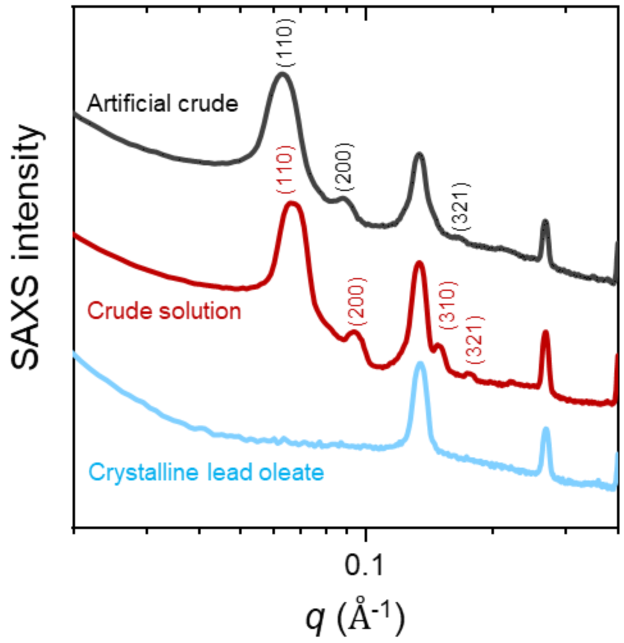

Figure S4. SAXS patterns comparing BCC NC SCs formed in the "artificial crude solution" versus the crude solution. The scattering pattern of crystalline $PbOA_2$ is there as references. These SAXS patterns were obtained at room temperature. All SAXS intensities are plotted as $I(q)$ in arbitrary units with logarithmic scaling of x- and y-axes.

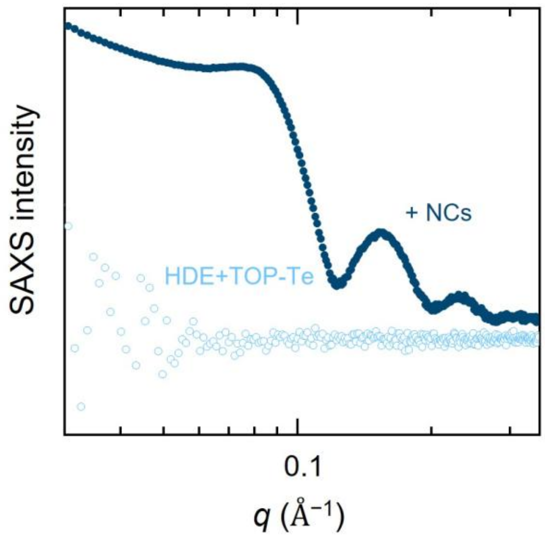

Figure S5. SAXS patterns of PbTe NCs dispersed in TOP-Te in HDE in the concentration estimated for the unreacted TOP-Te in the crude solution, considering a NC reaction yield of 20%. Scattering from HDE+TOP-Te without NC is shown for reference.

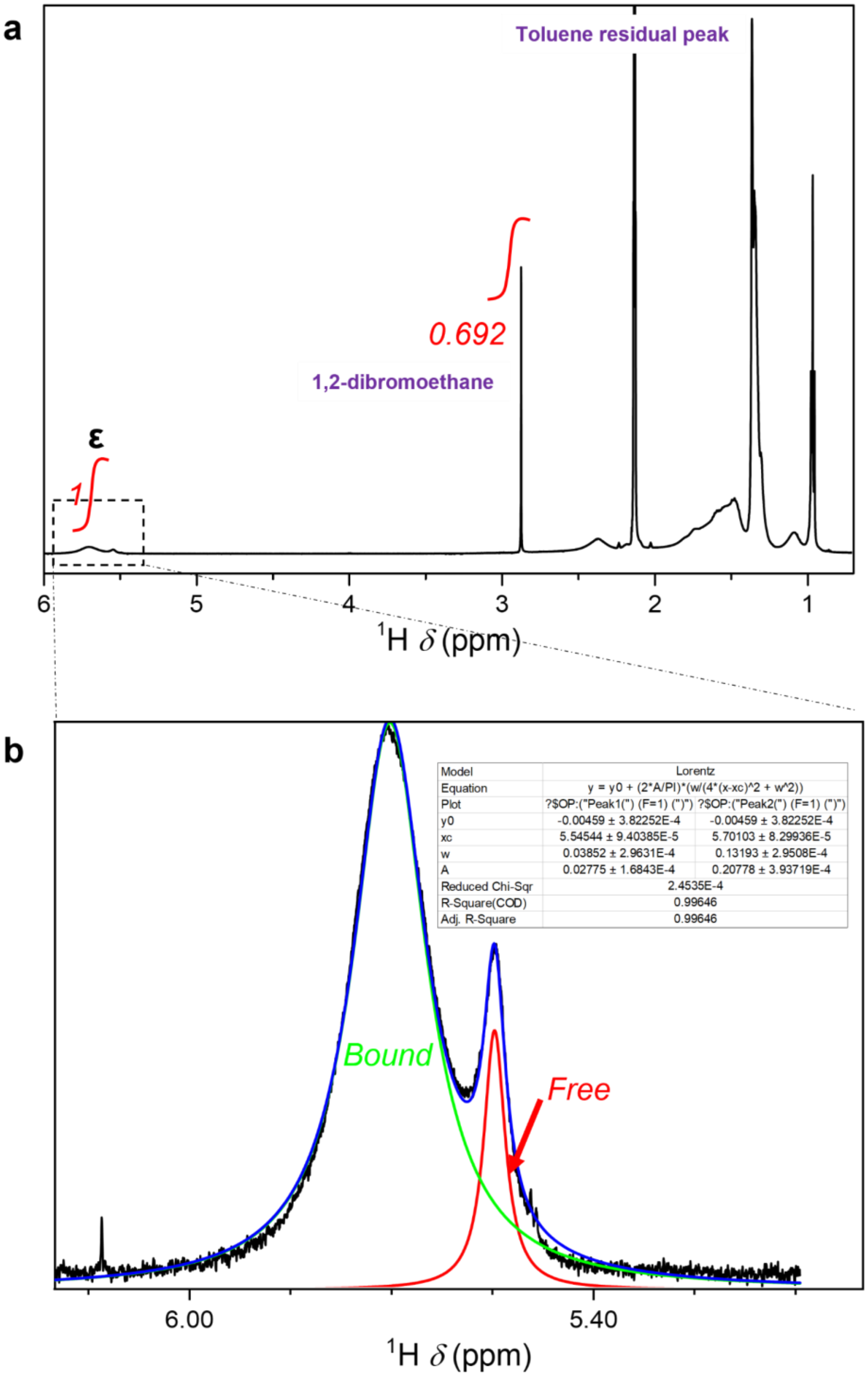

Figure S6. **(a)** Phase-corrected proton NMR spectrum of purified PbTe NCs dispersed in d-toluene. NC samples were prepared by isolating a defined amount of NC and redispersing them in d-toluene containing a known quantity of 1,2-dibromoethane as an internal quantitative standard. The integrated areas (∫) of vinyl proton resonances of lead oleate ligands were referenced to the 1,2-dibromoethane signal to quantify the total content of bound and free oleate. (**b**) Deconvolution

of the vinyl region using Lorentzian fits distinguishes bound (5.7 ppm) and free (5.5 ppm) oleate species. Analysis yields 88.5% bound oleate and 11.5% free oleate. Assuming spherical NCs, this corresponds to a ligand density of 3.9 oleate/nm^2. From the measured free oleate content, we estimate that the purified NC dispersion contains Pb-oleate oligomers. Further purification to remove the free oleate species leads to colloidally unstable PbTe NCs.

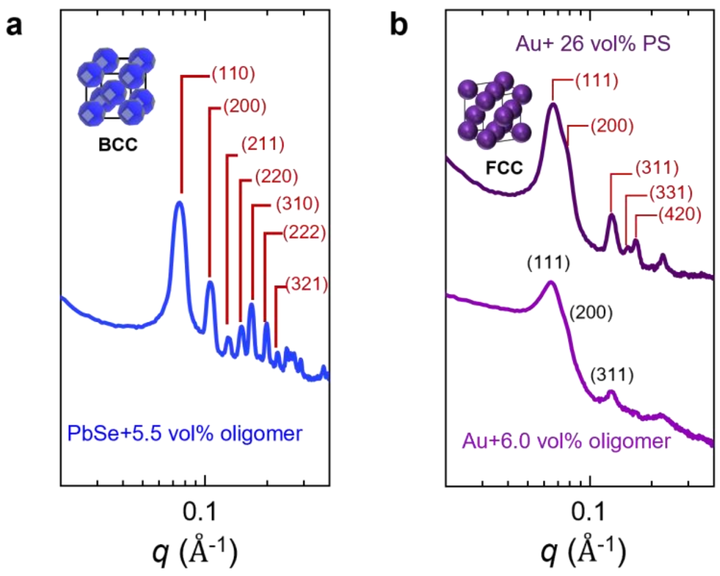

Figure S7. Structural characterization of depletion-derived SCs in reaction medium shown in Figures 3a,b. (**a**) SAXS pattern of a 6.8 nm PbSe NC crude reaction mixture containing 5.5 vol% Pb–oleate oligomers indexed to a BCC superlattice. (**b**) SAXS pattern of an 8.6 nm Au NC crude reaction mixture containing either 26 vol% polystyrene (PS) or 6.0 vol% Pb-oleate oligomers, indexed to a face-centered cubic (FCC) superlattice.

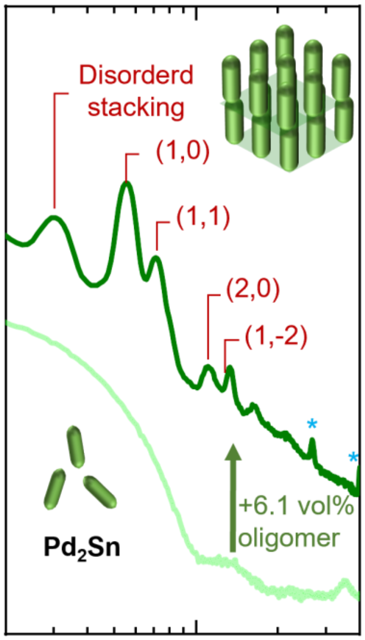

Figure S8. SAXS pattern of the Pd_2Sn nanorod assemblies obtained from mixing Pd_2Sn nanorods with Pb-oleate oligomer in HDE at 6.1 vol% concentration. Additional reflections marked in blue asterisks correspond to $PbOA_2$ precipitate. The peak positions indicate in-plane ordering into rhombic sheets and short-range ordering along the out-of-plane direction. Small deviations from square symmetry indicate local in-plane distortions (~80° angle between the lattice vectors). SAXS pattern of the crude solution without added depletant is shown for reference (light green).

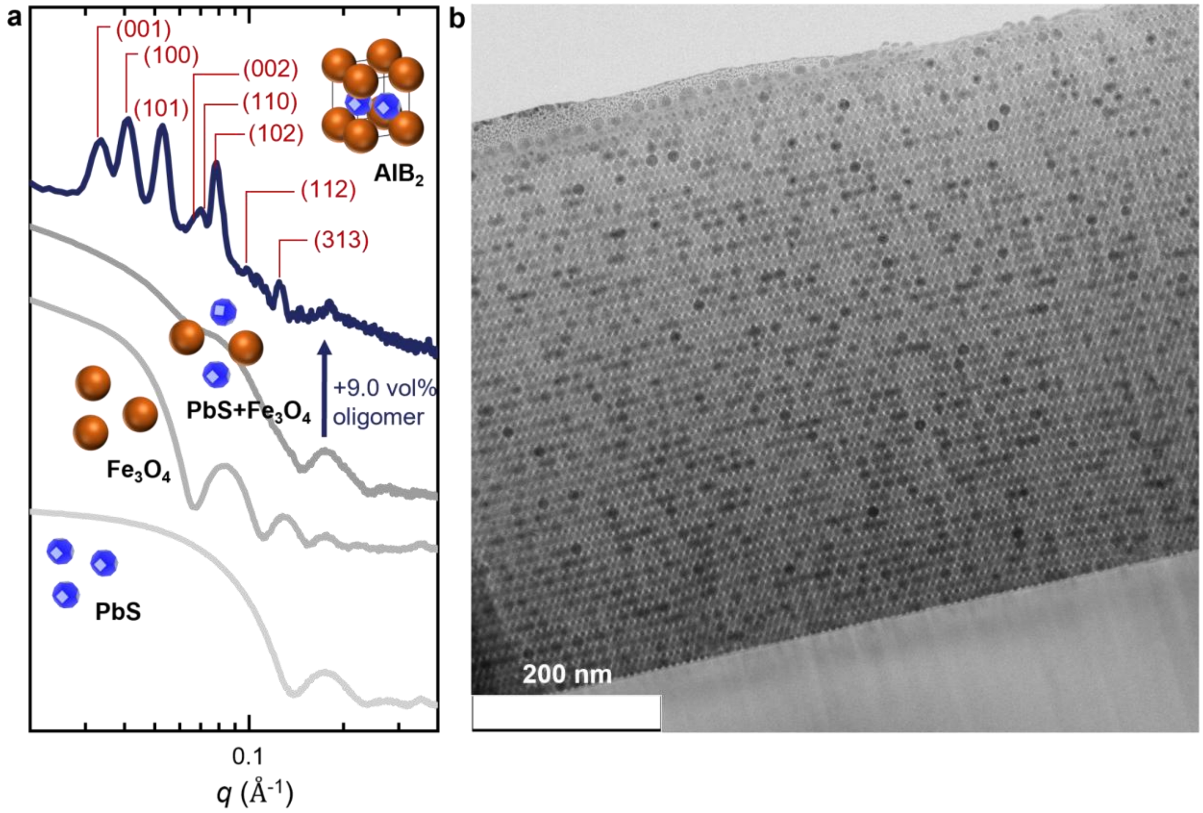

Figure S9. Structural characterization of binary PbS-Fe_3O_4 SCs. (**a**) SAXS pattern of PbS-Fe_3O_4 SCs obtained from a mixture of 6.5 nm PbS and 14 nm Fe_3O_4 colloidal NCs and 9.0 vol% Pb–oleate oligomer in toluene, indexed to the AlB_2 structure. SAXS patterns of the PbS and Fe_3O_4 NC dispersions and their mixture without added depletants are shown for reference (grey lines). (**b**) Cross-sectional TEM image of the binary SC, prepared by focused ion beam (FIB) milling using Cryo-FIB/SEM Aquilos 2 (Thermo Fisher Scientific) instrument, revealing the internal SC structure.

Supporting Note 1: Form factor of NCs

We obtained the size and shape of the PbTe NCs from SAXS patterns measured for a dilute dispersion of the purified particles in hexane. At this volume fraction, no interparticle correlations were detected in the accessed q range, hence the system was treated as a non-correlated "colloidal gas". We fit the data using an isotropic particle form factor of spheres with a lognormal size distribution. Instrumental resolution effects (broadening) were accounted for by convoluting the model intensity with a q-independent Gaussian resolution function with a full width at half maximum of 0.006 1/Å.

To account for atomic-scale surface disorder and small deviations from a sharp particle interface, the form factor was modeled using a "fuzzy sphere" model, in which the spherical form factor is multiplied by an interfacial width term $\exp(-q^2fz^2)$:[16]

$$I_{NP}(q, D, PD, fz, \phi) = \frac{\phi \Delta\rho^2}{< V >} f(q, 0, \sigma) \otimes P(q, D, PD, fz) \tag{1}$$

$$P(q, D, PD, fz) = \exp(-q^2 fz^2) \int_0^\infty w(r) \frac{9V(r)^2(\sin(qr) - qr * \cos(qr))^2}{(qr)^6} dr \tag{2}$$

Here Φ is the NC volume fraction, $\Delta\rho$ is the scattering length density contrast, D the mean diameter with polydispersity factor of PD, $w(r)$ is the probability density function of the size dispersion (~number fraction), $V(r)$ is the particle volume, fz is the surface fuzziness (the standard deviation of the interfacial width), and $f(q,0,\sigma)$ is a Gaussian resolution function with distribution with full width at half maximum of 0.006 1/Å. The particle diameter, relative polydispersity, volume fraction, constant background, and an interfacial width parameter were refined.

The resulting fits are shown in **Figure S10** and the corresponding fitting parameters are summarized in **Table S4**. The fuzzy sphere model gives a better-quality fit, as indicated by the

residual curve and the reduced chi-squared statistics. Interestingly, the fuzziness parameter (the characteristic range within which it is hard to tell where the surface is) converged to ~3 Å or ~1 atomic layer thickness, meaning a partially occupied shell of ~3 layers. While this latter fit was still not “perfect” (reduced $\chi^2 > 1$), this five-parameter model (diameter, polydispersity, surface disorder, volume fraction, background) is sufficient to reproduce the measured data with physically sensible parameters. We therefore applied it to the multicomponent samples as well.

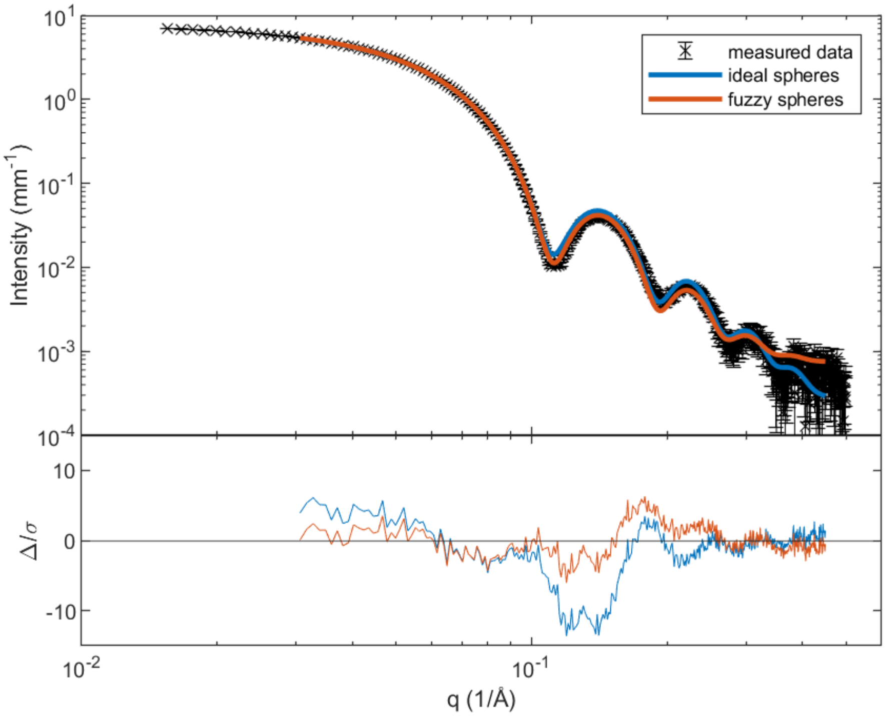

Figure S10. Measured scattering intensity (black markers) of a dilute PbTe NC dispersion in hexane, after background and buffer subtraction and path length normalization. Fits using ideal sphere (blue) and fuzzy sphere (red) models are shown. The lower panel shows the corresponding residuals.

Table S4. Fit parameters for the SAXS data shown in Figure S10 using homogeneous sphere and fuzzy sphere models.

	Ideal spheres	Fuzzy spheres
diameter (nm)	8.0	8.0
polydispersity	0.068	0.060
fuzziness STD (nm)	0	0.3
NC volume fraction	9.0×10^{-5}	9.3×10^{-5}
background (mm^{-1})	~0	0.0007
reduced χ^2	13.8	3.82

Supporting Note 2: Modeling SCs scattering

The SAXS patterns were modeled as a sum of distinct scattering contributions, including free NCs, ordered NC superlattices, solid $PbOA_2$, dissolved $PbOA_2$, and background. SC scattering was modeled using full profile approach analogous to powder diffraction analysis. The structure factor was described as a sum of Bragg reflections with positions and multiplicities determined by the assumed lattice symmetry (BCC in case of PbTe NCs).

The intensities of each reflection were given by the product of a common scaling factor, the multiplicity of the reflection, and a Lorentz correction term proportional to q^{-2}. Peak broadening was modelled as a combination of instrumental broadening and sample-related contribution. Instrumental broadening was described by a Gaussian instrumental profile with full width at half maximum of 0.006 1/Å, determined from measurements of a NIST LaB_6 standard and geometric simulations[17]. Sample-related broadening included Lorentzian contributions accounting for finite SC domain size (q-independent) and lattice strain (q-dependent). Size polydispersity was accounted as a lognormal size distribution in real space or, equivalently, as an additional Gaussian broadening contribution to the peak width.

The SC scattering intensity was expressed as the product of the NC form factor and the SC structure factor. The NC form factor parameters: particle size, polydispersity, and interfacial width, were defined as described in the previous section. The SC structure factor was described by 4 parameters: an overall intensity scale, the nearest-neighbor distance, the SC domain size, and the lattice strain.

$$I_{SC}(q, D, PD, fz, c, d_{NN}, \gamma_{size}, \gamma_{strain}) = \frac{c}{q^2} * P(q, D, PD, fz) * \sum_{hkl} m_{hkl} V(q, q_{hkl}(d_{NN}), \gamma_{size} + q^2\gamma_{strain}, \sigma_{IPF})$$

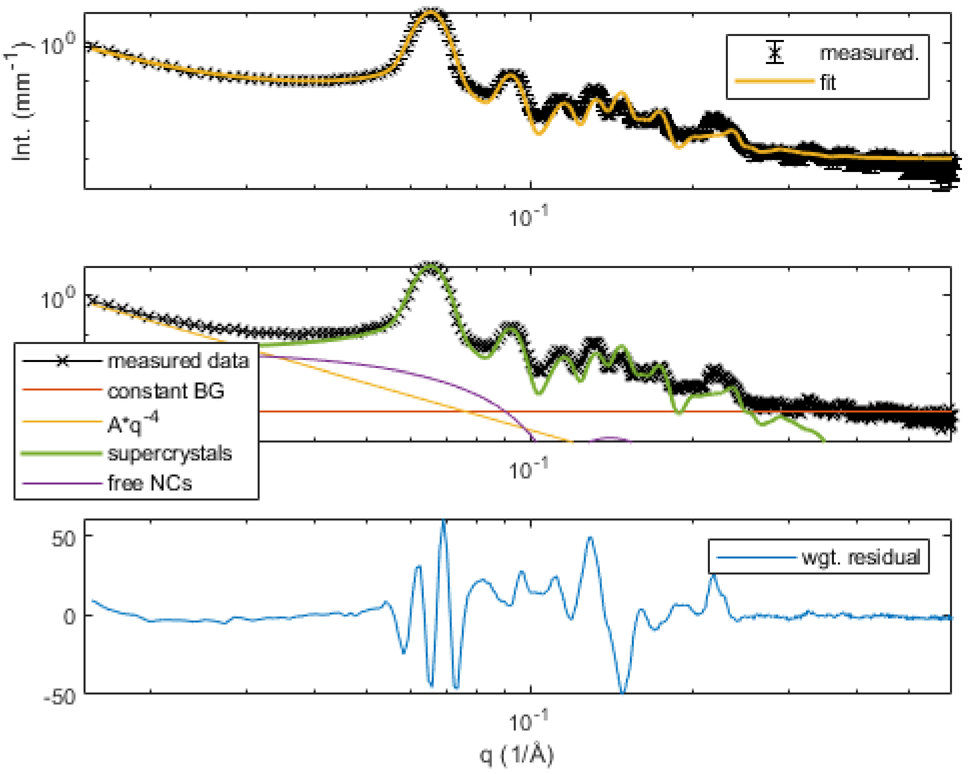

Figure S11. Measured SAXS intensity of purified PbTe SC (symbols) and corresponding fits obtained using the BCC lattice model (lines). The lower panel shows the weighted residuals of the fit.

The solid $PbOA_2$ phase was described as a set of equidistant reflections with pseudo-Voigt peak shapes and q-independent broadening, yielding an interlayer spacing of 4.65 nm, consistent with reported lead oleate lamellar structures[18,19]. The dissolved $PbOA_2$ contribution was modeled empirically using Lorentzian-like empirical correlation function with a $n = 3$ exponent and $L = 5.5$ Å based on a fit to the pure lead oleate solution[20].

$$I_{cluster}(q, A, L) = \frac{A}{1 + (qL)^3}$$

When multiple scattering contributions were present, the NC form factor parameters were fixed, and the remaining parameters describing the SC and other contributions were refined least-squares

fitting. The resulting fit parameters for the PbTe SC modeled assuming a BCC lattice are summarized in **Table S5** and the results of the modeling are shown in **Figure S11**.

Table S5. Fit parameters obtained from SAXS modeling of PbTe SCs assuming a BCC lattice, representative of the data shown in Figure S11.

Parameters	Free NCs	Crude solution	Purified SCs
NC diameter (nm)	8.0	8.0	8.0
NC polydispersity	0.06	0.06	0.06
NC fuzziness	3.0	3.0	3.0
Free NC volume fraction	9.28E-05	6.10E-06	4.96E-07
SC nearest neighbor distance (nm)	-	11.36	11.65
SC amplitude	-	1.18E-07	1.37E-07
SC domain size	-	32.6	47.8
Solid $PbOA_2$ spacing	-	46.7	-
Solid $PbOA_2$ amplitude	-	0.0056	-
Dissolved $PbOA_2$ amplitude	-	0.053	-
q^{-4} amplitude	-	2.02E-07	3.56E-08
Constant background	0.0015	0.0067	0.0011
Mean squared error	3.6	131	134

Supporting Note 3: Structure of Pb–oleate oligomers in HDE

$PbOA_2$ precipitates from HDE at room temperature, but forms clear and colorless solutions above 30 °C. Despite the optical clarity, SAXS patterns demonstrate that these solutions are not homogeneous at the molecular level. Instead, $PbOA_2$ forms supramolecular species under the conditions relevant for the NC synthesis and assembly.

SAXS data from $PbOA_2$ solutions containing an excess of OA were collected at 45 °C, revealing a weak but reproducible scattering feature centered around 0.12 $Å^{-1}$, at concentrations corresponding to the artificial crude solution. This feature weakens upon dilution and becomes more pronounced at higher concentrations, while the rest of the profile remains largely unchanged. These observations indicate the presence of stable nanoscale aggregates whose population scales with concentration.

To estimate the characteristic size and shape of the dispersed species, SAXS data from the most dilute solution were analyzed using standard form factor models implemented in SasView. Spherical models fail to reproduce the experimental data without invoking unphysical size dispersions. In contrast, anisotropic models such as ellipsoids and rectangular parallelepipeds yield good fits with reduced chi squared values close to unity. While the exact geometry cannot be uniquely determined, both successful models converge on aggregates with one short dimension on the order of 0.5 nm and lateral dimensions between approximately 1.5 and 3 nm (**Figure S12** and **Table S6**).

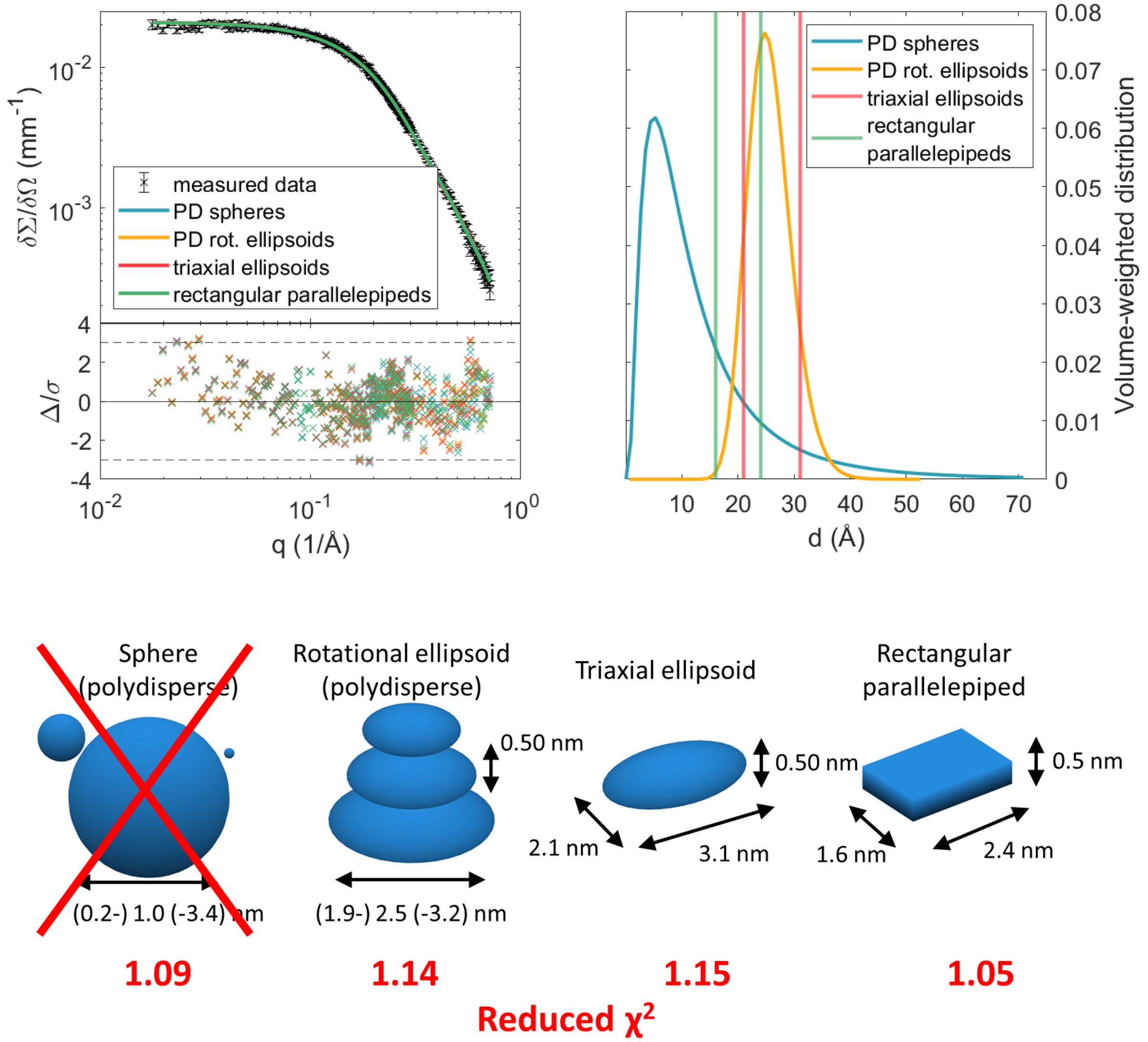

Figure S12. Top left: data measured on the most diluted solution and the fit curves, including the weighted residuals; top right: volume-weighted size dispersions for the polydisperse models, and the fit (lateral) parameters for the others; bottom: shape hypotheses for the dispersed Pb-oleate species, including the unlikely sphere model. The marked size ranges represent the volume-weighted 10%, 50%, and 90% quantiles of parameters with size dispersion; the red numbers are the reduced chi-squared statistics.

Table S6. Fit parameters for the most suitable 3 shape models (Figure S12): K intensity scaling factor, D edge length or diameter (single value or number-weighted mean of distribution), PD polydispersity as implemented in SasView.

Rotational ellipsoids with polydisperse equatorial radius		Triaxial ellipsoids		Rectangular parallelepipeds (rectangular prisms, "bricks")	
K	0.00031	K	0.00031	K	0.00029
D (short)	0.5 nm	D (short)	0.5 nm	D (short)	0.5 nm
Median D (long)	2.4 nm	D (medium)	2.1 nm	D (medium)	1.6 nm
PD (long)	0.15	D (long)	3.1 nm	D (long)	2.4 nm
Chi^2	1.14	Chi^2	1.15	Chi^2	1.05

The shortest dimension is more sensitive to background subtraction and instrumental limitations at high *q* values and therefore carries a larger uncertainty than the lateral dimensions. It should be interpreted as indicating a sub nanometer thickness rather than a precisely defined structural parameter. In contrast, the lateral dimensions are robust across different form factor models and concentrations and consistently fall in the range of approximately 1.5 to 3 nm. We refer to these aggregates as Pb-oleate oligomers. Importantly, the structure of the solution is strongly affected by the excess oleic acid. Pure lead-oleate measured in the same condition show a broad, featureless scattering pattern (see Figure 2b in the main text) indicative of strong aggregation beyond this few nm size range.

Metal carboxylates are known to form inverse micelles in apolar, non-coordinating solvents.[21] The size of these aggregates is under debate, with reported numbers ranging from few atoms to hundreds of nanometers.[22,23] Our results indicate that in presence of excess oleic acid, lead-oleate forms oligomers with tens of lead atoms, with relatively low size dispersion.

Having established the characteristic size and anisotropy of the Pb-oleate oligomers at low concentration, we next examined their behavior at higher concentrations (**Figure S13** and **Table S7**). In this regime, the SAXS profiles exhibit clear interparticle correlations. These data can be described by combining the oligomer form factor with a hard sphere structure factor. The extracted effective interaction diameter is approximately 3.2 nm, comparable to the NC center-to-center distances observed in the SCs. These results are in line with the DOSY-based hydrodynamic radius[23] and SANS-based shell outer diameter.[24] Importantly, the oligomer dimensions remain nearly constant across the studied concentration range, indicating that these species are structurally stable against dilution. It is important to note that the dimensions obtained for the Pb-oleate oligomers are on the relevant length scale to generate depletion interactions. All data and fit curves are shown in **Figure S13**, and the fit parameters are listed in **Table S7**.

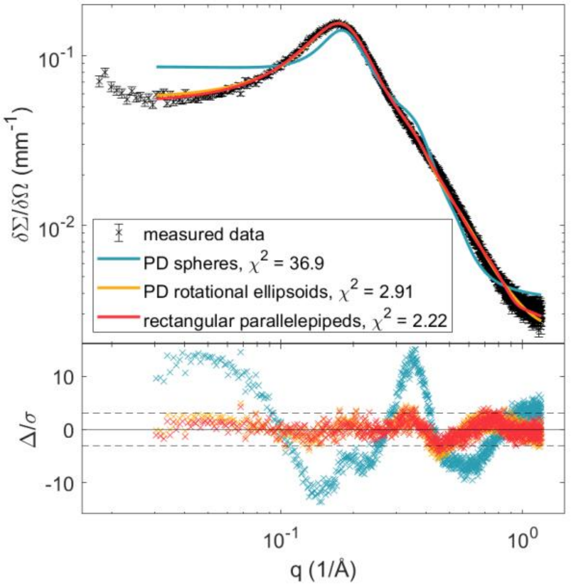

Figure S13. Confirmation of the particle shape using the high concentration data and two of the tested models after including hard sphere repulsion.

Table S7. Fit parameters from three different concentration solutions (Figure S13) using the parallelepiped model with hard sphere repulsion; the less reliable interaction parameters from the medium concentration solution are shown in parentheses.

Fit parameter	1.8 vol%	6.0 vol%	18 vol%
Short edge	0.5 nm	0.5 nm	0.5 nm
Medium edge	1.6 nm	1.5 nm	1.4 nm
Long edge	2.4 nm	2.2 nm	2.1 nm
Effective diameter (including ligands)	-	(3.6 nm)	3.2 nm
Volume fraction (including ligands)	-	(0.05)	0.19

To provide a chemically plausible structural picture of these oligomers, we constructed atomistic models of lead carboxylate fragments. Starting from the reported crystal structure of lead stearate,[25] atoms beyond the alpha carbon were removed and nanoscale fragments with lateral dimensions of approximately 2 nm were generated (**Figure S14**). Scattering patterns calculated using the Debye equation for such fragments reproduce the main features of the experimental SAXS data. Such structures are also in line with previous reports that structure of dissolved lead-carboxylates resemble the (lamellar phase of the) corresponding pure compound.[22,23] These atomistic models are intended to illustrate that nanometer-sized fragments of Pb-O carboxylate sheets are consistent with the experimentally observed form factor. They should not be interpreted as unique or definitive structural solutions.

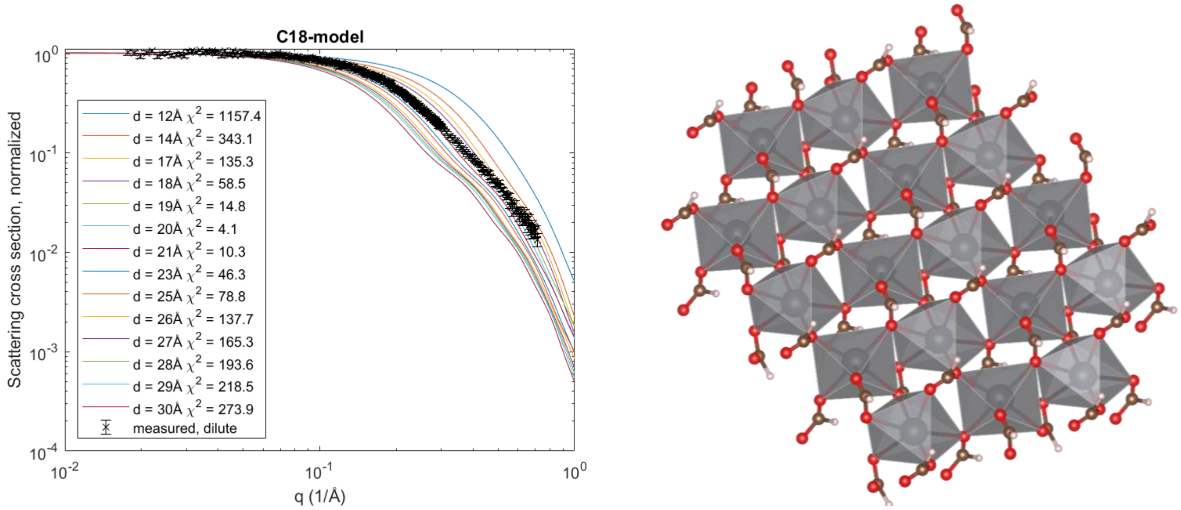

Figure S14. Left: scattering patterns of a Pb-carboxylate nanosheets with different shapes and sizes; right: the 19 Å and 20 Å models (grey: Pb, red: O, brown: alpha-C atoms, white: beta-C of ligands, which are not included in the calculations). The visualization and handling of the crystal structures was done using VESTA.[26] The cluster (right) contains 18 lead atoms.

To visualize a solvated and relaxed ligand shell around such fragments, we further generated a representative model by replacing stearate chains with oleate and relaxing the ligand conformations using molecular dynamics simulations. During the simulation, the inorganic Pb–O core and alpha carbon atoms were kept rigid, while the remaining carbon atoms were treated using a unified atom representation. The simulations were performed in LAMMPS[27] using previously reported potential parameters[28] and run for 60 ns with a 1 fs time step. The resulting relaxed ligand conformations were used exclusively for visualization purposes, with final structural images generated using Avogadro and VESTA.[26]

Supporting Note 4: Thermal stability of Pb–oleate oligomers under reaction conditions

To assess the stability of Pb–oleate oligomers across the temperature range relevant for NC growth and assembly, temperature-dependent SAXS measurements were performed on the two higher concentration solutions. Scattering profiles were collected between 30 and 160 °C (**Figure S15**). Over this temperature range, no phase transitions or substantial changes in the scattering profile are observed. All data can be described using the same anisotropic form factor combined with a hard sphere structure factor, demonstrating that the identity of the dispersed species remains unchanged upon heating.

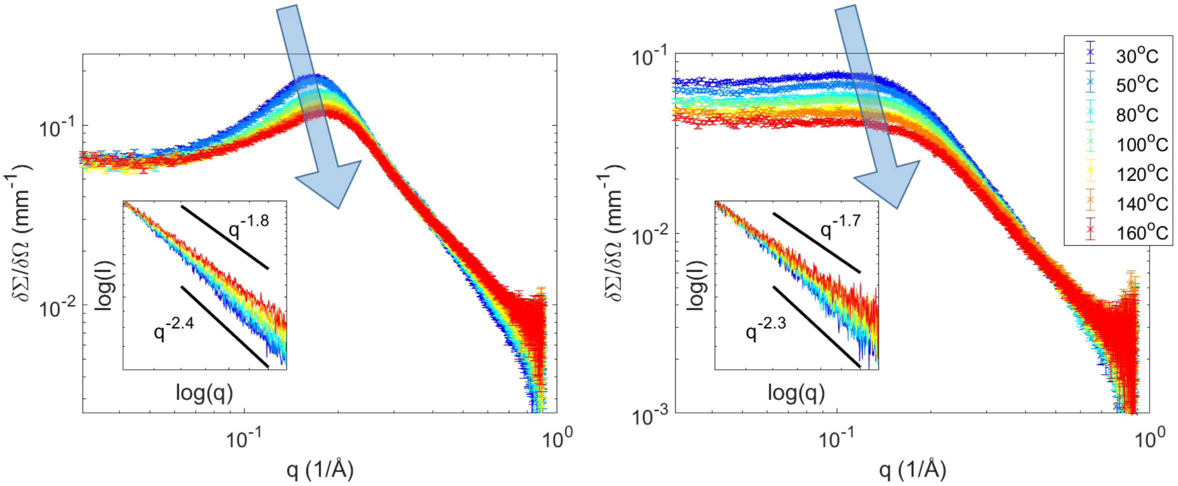

Figure S15. Data measured on the 18 vol% (left) and 6 vol% (right) lead oleate solution; the arrow indicates scattering profiles of the respective solution during heating, showing the trend in the correlation peak stemming from decreasing effective diameter and the corresponding volume fraction. Both SAXS data are thermally reversible, while they do not exhibit any molecular ordering during cooling.

With increasing temperature, gradual shifts in the position and intensity of the correlation peak are observed. These trends are consistent with a reduction in effective particle–particle interactions, reflected in a decrease of the effective interaction diameter and volume fraction. Such changes are

expected from increased thermal motion and modifications of solvation and do not indicate a change in the oligomeric nature of the species. Importantly, the characteristic form factor features associated with the nanoscale oligomers persist throughout the full temperature window and are fully reversible upon cooling.

Supporting Note 5: Determination of order-disorder transition temperatures

Thermally reversible superlattice formation was investigated by temperature-dependent SAXS. SAXS profiles were recorded during controlled heating and cooling cycles for NC solutions containing different volume fractions of Pb–oleate oligomers in HDE and toluene. Scattering profiles were collected every 150 s while heating from room temperature to the target temperature range using heating rates between 1 and 5 °C·min^{-1}. Slower heating rates were applied in the temperature region where changes in superlattice scattering occurred to ensure accurate determination of the order disorder transition. During cooling back to the initial temperature, SAXS patterns were recorded using ramp rates between 1 and 2 °C·min^{-1}.

Order-disorder transition temperatures were determined from the temperature-dependent scattering intensity associated with dispersed NCs and SCs. The total scattering intensity was integrated over q ranges corresponding to the characteristic NC peak ($0.054 < q < 0.062$ Å^{-1}) and SC peak ($0.072 < q < 0.080$ Å^{-1}). The total integrated intensities are denoted as $\Sigma\, NC$ and $\Sigma\, SC$, respectively.

A normalized relative scattering intensity was calculated as $I_{normal} = \frac{\sum NC - \sum SC}{\sum SC + \sum NC}$. Since this quantity can take negative values depending on the dominant scattering contribution, it was re-normalized from zero to one, $I'_{normal} = \frac{I_{normal} - I_{normal,min}}{I_{normal,max} - I_{normal,min}}$, where zero corresponds to the SC-dominant region. The normalized relative scattering intensity, I'_{normal}, was plotted as a function of temperature. Order-disorder transition temperatures were determined by linear interpolation between the superlattice dominant regime and the transition region. All data processing and visualization were performed using MATLAB and XSACT suite. Representative plots are shown in **Figures S16–S18**.

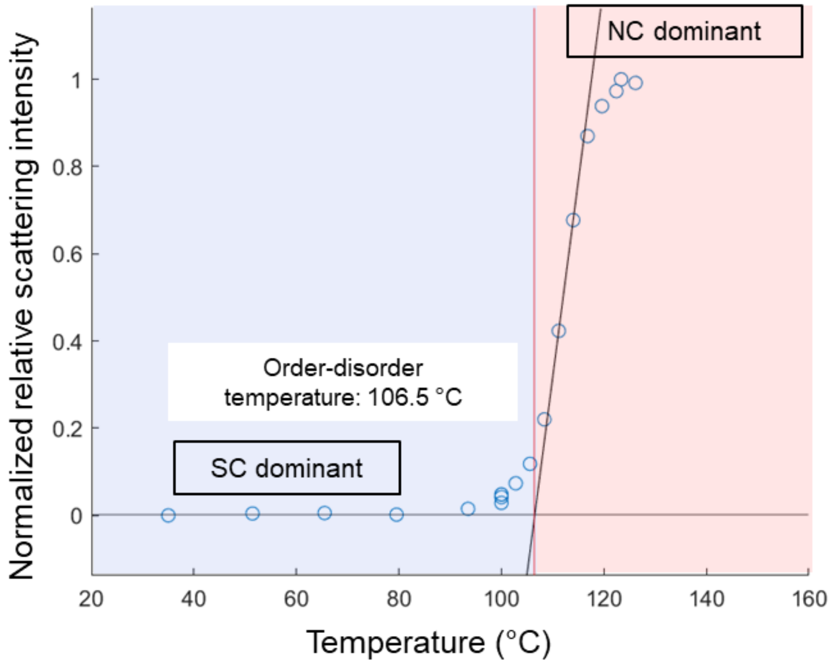

Figure S16. Order-disorder transition temperature for the 6.0 vol% Pb–oleate solution with 6.3 nm PbTe NCs (Figure 3c), determined from the intersection of two linear fits: the saturated SC region (blue) and the interpolated transition region (red).

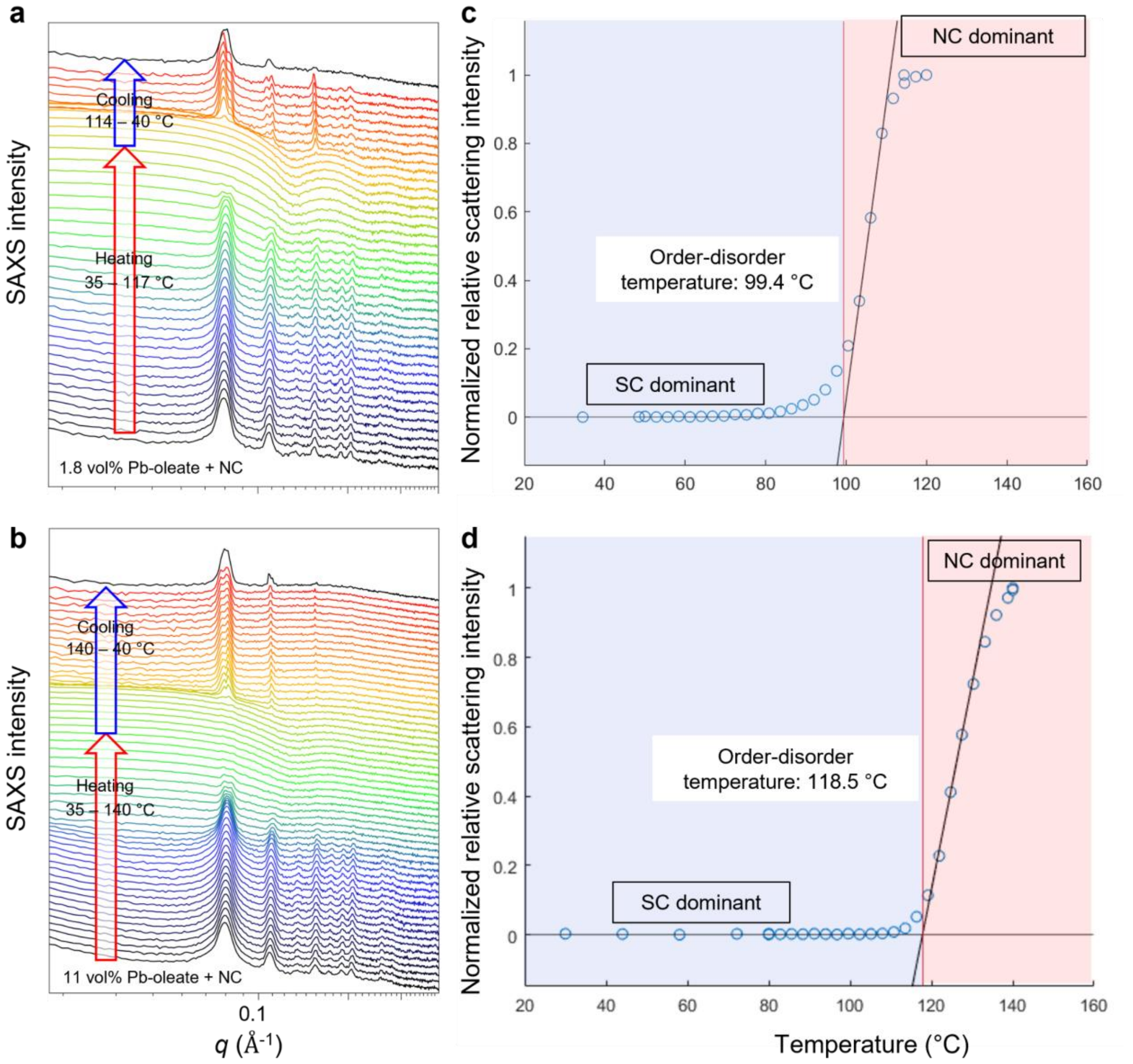

Figure S17. (**a**, **b**) Temperature dependent SAXS profiles of control mixtures containing 6.3 nm PbTe NCs with 1.8 vol% (**a**) and 11 vol% (**b**) Pb–oleate oligomer concentration in HDE, shown in Figure 3d. The preformed SCs at room temperature gradually dissolve into dispersed state upon heating after which the NCs recrystallized into BCC superlattices during cooling. (**c**, **d**) Order-disorder temperatures were extrapolated by linear interpolation of the normalized SAXS peak ratio of NC to SC to the baseline (SC dominant region), yielding 99.4 °C (**c**) and 118.5 °C (**d**), respectively.

Beyond thermal energy scale, temperature can also introduce secondary effects. SAXS reveals a modest reduction in oligomer size at elevated temperature (Figure S15), which could slightly

weaken depletion attraction. However, this change is small and does not dominate the observed transition behavior.

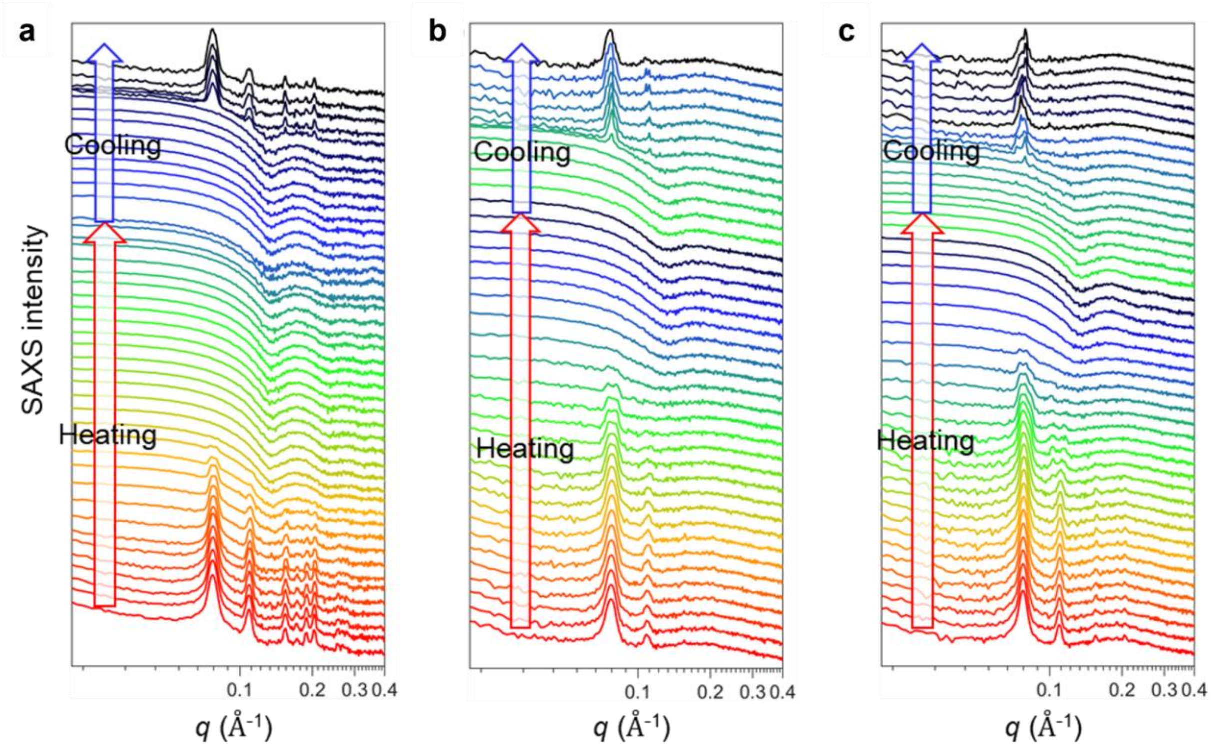

Figure S18. Temperature-dependent SAXS profiles of control mixtures containing 6.3 nm NCs with 1.8 vol% (**a**), 7.7 vol% (**b**), and 15 vol% (**c**) Pb–oleate oligomer concentration in toluene, shown in Figure 3d. Compared to Figure S17, the preformed SCs gradually dissolve into a dispersed state at lower temperatures, after which the NCs recrystallize into BCC superlattices during cooling. Order-disorder transition temperatures were extrapolated by linear interpolation of the normalized SAXS peak ratio of NC to SC to the baseline (SC dominant region), yielding 52.5 °C for 1.8 vol%, 67.6 °C for 7.7 vol%, and 78.7 °C for 15 vol%.

Setup for special SAXS measurements

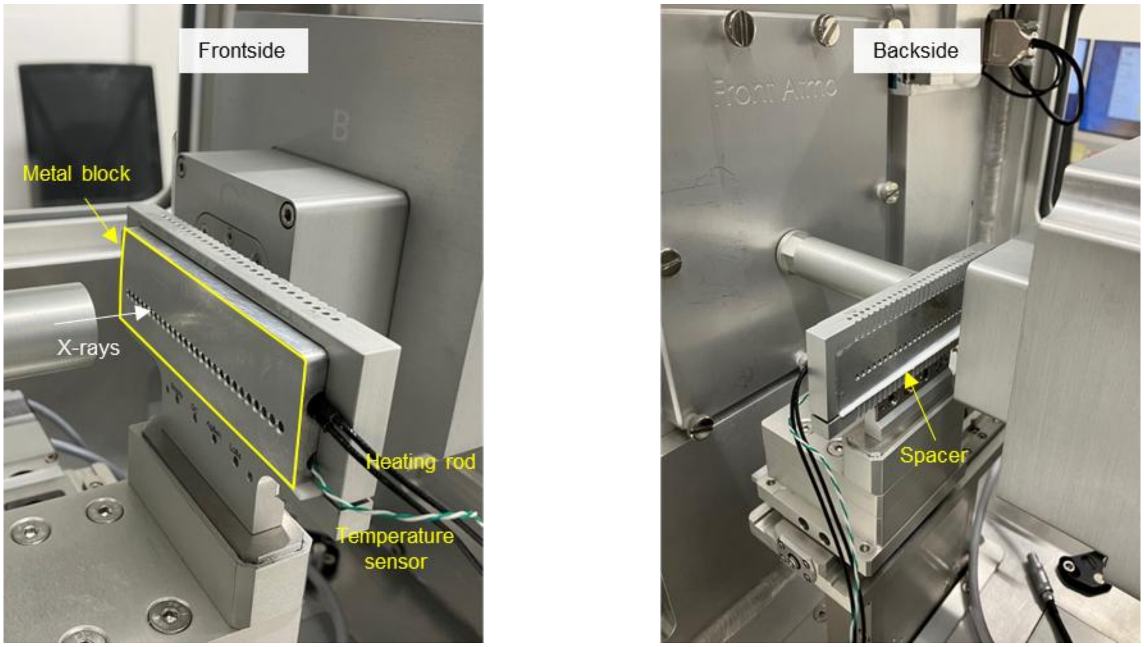

Figure S19. Photographs of the experimental setup used for temperature-dependent SAXS measurements. A multi-capillary heating stage installed inside the SAXS measurement chamber is shown from the front view (left) and back view (right). The heating stage consist of a heating rod inserted into a metallic block and a temperature sensor; both connected to a temperature controller (Digi-Sense TC9100 Advanced). A PTFE spacer was incorporated to serve as a thermal insulator to prevent heat transfer to the electronic components of the SAXS instrument. All measurements were carried out under ambient atmosphere.

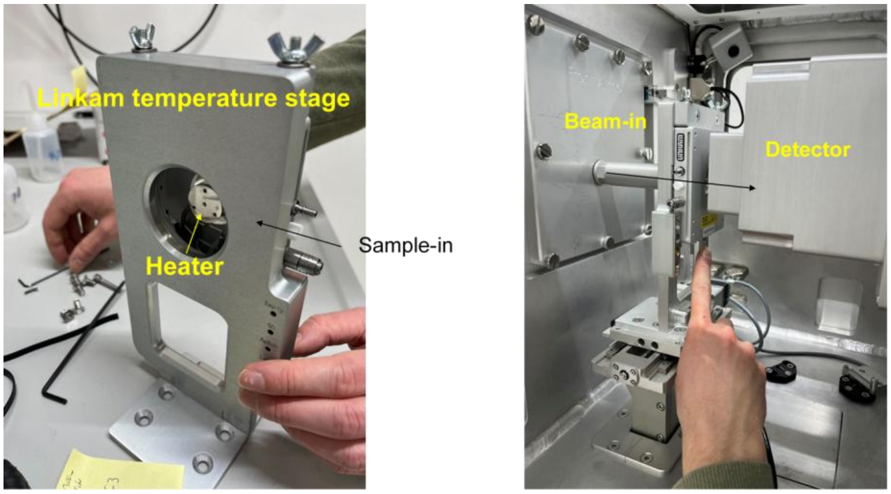

Figure S20. Linkam temperature stage used for temperature-dependent SAXS measurements. The photographs show the front view (left) and the side view (right) of the Linkam HFSX350 stage in the measurement chamber. All measurements were carried out under ambient atmosphere.

References

(1) Hendricks, M. P.; Campos, M. P.; Cleveland, G. T.; Jen-La Plante, I.; Owen, J. S. A tunable library of substituted thiourea precursors to metal sulfide nanocrystals. *Science* **2015**, *348* (6240), 1226-1230. DOI: 10.1126/science.aaa2951.

(2) Ibáñez, M.; Zamani, R.; Gorsse, S.; Fan, J.; Ortega, S.; Cadavid, D.; Morante, J. R.; Arbiol, J.; Cabot, A. Core–Shell Nanoparticles As Building Blocks for the Bottom-Up Production of Functional Nanocomposites: PbTe–PbS Thermoelectric Properties. *ACS Nano* **2013**, *7* (3), 2573-2586. DOI: 10.1021/nn305971v.

(3) Balazs, D. M.; Dunbar, T. A.; Smilgies, D.-M.; Hanrath, T. Coupled Dynamics of Colloidal Nanoparticle Spreading and Self-Assembly at a Fluid–Fluid Interface. *Langmuir* **2020**, *36* (22), 6106-6115. DOI: 10.1021/acs.langmuir.0c00524.

(4) Rupich, S. M.; Shevchenko, E. V.; Bodnarchuk, M. I.; Lee, B.; Talapin, D. V. Size-Dependent Multiple Twinning in Nanocrystal Superlattices. *J. Am. Chem. Soc.* **2010**, *132* (1), 289-296. DOI: 10.1021/ja9074425.

(5) Nafria, R.; Luo, Z.; Ibáñez, M.; Martí-Sànchez, S.; Yu, X.; de la Mata, M.; Llorca, J.; Arbiol, J.; Kovalenko, M. V.; Grabulosa, A.; et al. Growth of Au–Pd_2Sn Nanorods via Galvanic Replacement and Their Catalytic Performance on Hydrogenation and Sonogashira Coupling Reactions. *Langmuir* **2018**, *34* (36), 10634-10643. DOI: 10.1021/acs.langmuir.8b02023.

(6) Park, J.; An, K.; Hwang, Y.; Park, J.-G.; Noh, H.-J.; Kim, J.-Y.; Park, J.-H.; Hwang, N.-M.; Hyeon, T. Ultra-large-scale syntheses of monodisperse nanocrystals. *Nat. Mater.* **2004**, *3* (12), 891-895. DOI: 10.1038/nmat1251.

(7) Wu, B. H.; Yang, H. Y.; Huang, H. Q.; Chen, G. X.; Zheng, N. F. Solvent effect on the synthesis of monodisperse amine-capped Au nanoparticles. *Chinese Chem. Lett.* **2013**, *24* (6), 457-462. DOI: 10.1016/j.cclet.2013.03.054.

(8) Goubet, N.; Tempra, I.; Yang, J.; Soavi, G.; Polli, D.; Cerullo, G.; Pileni, M. P. Size and nanocrystallinity controlled gold nanocrystals: synthesis, electronic and mechanical properties. *Nanoscale* **2015**, *7* (7), 3237-3246, 10.1039/C4NR06513A. DOI: 10.1039/C4NR06513A.

(9) Doucet, M.; Adams, M.; Agouzal, N.; Alina, G.; Attala, Z.; Backman, M.; Bakker, J.; Beaucage, P.; Berger, J.; Bourne, R.; et al. *SasView Version 6.1.0*. 2025. http://doi.org/10.5281/zenodo.15775666 (accessed 2026-01-30).

(10) Bergström, L. Hamaker constants of inorganic materials. *Adv. Colloid Interface Sci.* **1997**, *70*, 125-169. DOI: https://doi.org/10.1016/S0001-8686(97)00003-1.

(11) Norihiro Suzuki, N. S.; Sadao Adachi, S. A. Optical Properties of PbTe. *Jpn. J. Appl. Phys.* **1994**, *33* (1R), 193. DOI: 10.1143/JJAP.33.193.

(12) Palik, E. D. *Handbook of Optical Constants of Solids*; Academic Press, San Diego, CA USA, 1998.

(13) Henke, B. L.; Gullikson, E. M.; Davis, J. C. X-Ray Interactions: Photoabsorption, Scattering, Transmission, and Reflection at E = 50-30,000 eV, Z = 1-92. *Atomic Data and Nuclear Data Tables* **1993**, *54* (2), 181-342. DOI: https://doi.org/10.1006/adnd.1993.1013.

(14) Parsegian, V.; Ninham, B. Application of the Lifshitz theory to the calculation of van der Waals forces across thin lipid films. *Nature* **1969**, *224* (5225), 1197-1198.

(15) Bishop, K. J.; Wilmer, C. E.; Soh, S.; Grzybowski, B. A. Nanoscale forces and their uses in self-assembly. *Small* **2009**, *5* (14), 1600-1630. DOI: 10.1002/smll.200900358.

(16) Stieger, M.; Pedersen, J. S.; Lindner, P.; Richtering, W. Are Thermoresponsive Microgels Model Systems for Concentrated Colloidal Suspensions? A Rheology and Small-Angle Neutron Scattering Study. *Langmuir* **2004**, *20* (17), 7283-7292. DOI: 10.1021/la049518x.

(17) Smilgies, D. M. Scherrer grain-size analysis adapted to grazing-incidence scattering with area detectors (vol 42, pg 1030, 2009). *J. Appl. Crystallogr.* **2013**, *46*, 286-286. DOI: 10.1107/S0021889812050054.

(18) Wang, Z.; Schliehe, C.; Bian, K.; Dale, D.; Bassett, W. A.; Hanrath, T.; Klinke, C.; Weller, H. Correlating superlattice polymorphs to internanoparticle distance, packing density, and surface lattice in assemblies of PbS nanoparticles. *Nano Lett.* **2013**, *13* (3), 1303-1311. DOI: 10.1021/nl400084k.

(19) Huang, X.; Zhu, J.; Ge, B.; Gerdes, F.; Klinke, C.; Wang, Z. In Situ Constructing the Kinetic Roadmap of Octahedral Nanocrystal Assembly Toward Controlled Superlattice Fabrication. *J. Am. Chem. Soc.* **2021**, *143* (11), 4234-4243. DOI: 10.1021/jacs.0c12087.

(20) *NIST IGOR/DANSE, Correlation_length*. Sasview.org, 2010. https://www.sasview.org/docs/user/models/correlation_length.html (accessed 2025-11-03).

(21) Pilpel, N. Properties of Organic Solutions of Heavy Metal Soaps. *Chem. Rev.* **1963**, *63* (3), 221-234. DOI: 10.1021/cr60223a001.

(22) Burrows, H. D.; Miguel, M. d. G.; Pereira, R. P. C.; Proença, N. M. B.; Cardoso, S. M. C.; Geraldes, C. F. G. C.; Gil, M. H.; Brown, W. Solution behaviour of lead(II) carboxylates in organic solvents. *Colloids Surf. A Physicochem. Eng. Asp.* **2004**, *250* (1), 459-465. DOI: https://doi.org/10.1016/j.colsurfa.2004.06.039.

(23) Cass, L. C.; Malicki, M.; Weiss, E. A. The chemical environments of oleate species within samples of oleate-coated PbS quantum dots. *Anal. Chem.* **2013**, *85* (14), 6974-6979. DOI: 10.1021/ac401623a.

(24) Abecassis, B.; Greenberg, M. W.; Bal, V.; McMurtry, B. M.; Campos, M. P.; Guillemeney, L.; Mahler, B.; Prevost, S.; Sharpnack, L.; Hendricks, M. P.; et al. Persistent nucleation and size dependent attachment kinetics produce monodisperse PbS nanocrystals. *Chem. Sci.* **2022**, *13* (17), 4977-4983. DOI: 10.1039/d1sc06134h.

(25) Martinez-Casado, F. J.; Ramos-Riesco, M.; Rodriguez-Cheda, J. A.; Redondo-Yelamos, M. I.; Garrido, L.; Fernandez-Martinez, A.; Garcia-Barriocanal, J.; da Silva, I.; Duran-Olivencia, M.; Poulain, A. Lead(ii) soaps: crystal structures, polymorphism, and solid and liquid mesophases. *Phys. Chem. Chem. Phys.* **2017**, *19* (26), 17009-17018. DOI: 10.1039/c7cp02351k.

(26) Momma, K.; Izumi, F. VESTA 3 for three-dimensional visualization of crystal, volumetric and morphology data. *J. Appl. Crystallogr.* **2011**, *44* (6), 1272-1276. DOI: doi:10.1107/S0021889811038970.

(27) Thompson, A. P.; Aktulga, H. M.; Berger, R.; Bolintineanu, D. S.; Brown, W. M.; Crozier, P. S.; in 't Veld, P. J.; Kohlmeyer, A.; Moore, S. G.; Nguyen, T. D.; et al. LAMMPS - a flexible simulation tool for particle-based materials modeling at the atomic, meso, and continuum scales.

Computer Physics Communications **2022**, *271*, 108171. DOI: https://doi.org/10.1016/j.cpc.2021.108171.

(28) Winslow, S. W.; Swan, J. W.; Tisdale, W. A. The Importance of Unbound Ligand in Nanocrystal Superlattice Formation. *J. Am. Chem. Soc.* **2020**, *142* (21), 9675-9685. DOI: 10.1021/jacs.0c01809.
